# A world review of the bristle fly parasitoids of webspinners

**DOI:** 10.1186/s40850-022-00116-x

**Published:** 2022-07-04

**Authors:** Davide Badano, Alice Lenzi, James E. O’Hara, Kelly B. Miller, Andrea Di Giulio, Filippo Di Giovanni, Pierfilippo Cerretti

**Affiliations:** 1 Museum of Zoology, Sapienza University of Rome, Piazzale Valerio Massimo 6, 00162 Rome, Italy; 2 Department of Biology and Biotechnologies “Charles Darwin”, Sapienza University of Rome, Piazzale A. Moro 5, 0, 0185 Rome, Italy; 3 Canadian National Collection of Insects, Agriculture and Agri-Food Canada, Ottawa, Ontario K1A 0C6 Canada; 4 Department of Biology and Museum of Southwestern Biology, University of New Mexico, Albuquerque, NM 87131 USA; 5 Department of Science, RomaTre University, Viale G. Marconi 446, 00146 Rome, Italy; 6 Department of Life Sciences, University of Siena, Via A. Moro 2, 53100 Siena, Italy

**Keywords:** Diptera, Embioptera, New species, Parasitoids, Polyneoptera, Tachinidae, Trophic shift

## Abstract

**Background:**

Dipteran parasitoids of Embioptera (webspinners) are few and extremely rare but known from all biogeographical regions except Australasia/Oceania. All belong to the fly family Tachinidae, a hyperdiverse and widespread clade of parasitoids attacking a variety of arthropod orders.

**Results:**

The webspinner-parasitizing Diptera are reviewed based mostly on records from the collecting and rearing by Edward S. Ross. A new genus is erected to accommodate a new Afrotropical species, *Embiophoneus rossi* gen. et sp. nov. The genus *Perumyia* Arnaud is reviewed and a new species, *Perumyia arnaudi* sp. nov., is described from Central America while *P. embiaphaga* Arnaud is redescribed and new host records are given. A new species of *Phytomyptera* Rondani, *P. woodi* sp. nov., is described from Myanmar, representing the first report of a member of this genus obtained from webspinners. The genus *Rossimyiops* Mesnil is reviewed, *R. longicornis* (Kugler) is redescribed and *R. aeratus* sp. nov., *R. fuscus* sp. nov. and *R. rutilans* sp. nov. are newly described from the Oriental Region, and an updated key to species is given.

**Conclusions:**

Webspinners were probably colonized independently at least four times by tachinids shifting from other hosts, most likely Lepidoptera.

**Supplementary Information:**

The online version contains supplementary material available at 10.1186/s40850-022-00116-x.

## Background

Webspinners—Embioptera, Embiodea or Embiidina [1]—are an ancient group of insects that date back to the late Permian, according to the fossil-calibrated insect phylogeny by Montagna et al. [2]. Present-day embiopterans are found at low to mid latitudes with their greatest diversity in tropical parts of the world [3, 4]. The approximately 400 described species belong to about 90 genera and 13 families [1, 3, 5] and the order itself belongs to the Polyneoptera, where it is probably the sister taxon of either the Phasmatodea or the Zoraptera [2–10]. Ross [11, 12] estimated that the actual number of species could be as high as 2000.

Webspinners are among the least collected insects largely because of their cryptic lifestyle. As their common name implies, these insects—adults as well as nymphs—produce silk using glands in their protarsi and spin retreats or, in some species, elaborate maze-like galleries in which they spend virtually their entire lives. They are herbivorous, lichenophagous or detritivorous and construct their galleries on the bark of trees, under stones, in leaf litter, and in other places that afford them access to such food sources as lichens, moss, bark and dead leaves [4, 11]. The galleries of webspinners are mostly inhabited by neotenic females and their nymphs. Males of most groups do not feed and are usually short-lived, in most groups dispersing quickly from their home galleries after emerging as adults in search of females with which to mate. These circumstances have hampered the taxonomic study of webspinners because the neotenic females possess fewer diagnostic characters than males, yet males are rarely captured in galleries. The much-coveted males are most readily obtained by rearing from collected nymphs, from eggs obtained from fertilized females or by catching them at lights at night [12, 13].

A major contributor to our knowledge of the Embioptera was Edward S. Ross (1915–2016), who published on this group over a period of approximately 70 years. He was an avid collector who travelled the world in search of webspinners. He eventually amassed a collection of about 350,000 specimens housed in the California Academy of Sciences in San Francisco (CAS) [14]. On very rare occasions Ross reared dipteran parasitoids from his live cultures of webspinners, all of them belonging to the family Tachinidae. These specimens were pinned and are also housed in CAS. The reared tachinids, 21 in number, were borrowed by us and form the basis for this taxonomic review.

The Tachinidae are a major clade of calyptrate Diptera with ca. 8500 described species [15, 16], all developing as endoparasitoids of at least 15 orders of arthropods including Lepidoptera (~ 60% of host species), Coleoptera (~ 15%), Heteroptera (~ 13%), Hymenoptera (~ 6%), Polyneoptera (Embioptera, Dictyoptera, Orthoptera, Phasmatodea) and even centipedes and scorpions [17, 18]. Only three species are currently known to parasitize webspinners: *Perumyia embiaphaga* Arnaud in Peru [19], *Rossimyiops exquisitus* (Richter) in Iran and Yemen, and *Rossimyiops whiteheadi* Mesnil in South Africa [20]. To these are added below a new monotypic genus from Africa, a new species of *Perumyia* Arnaud from Mexico and Nicaragua, a new species of *Phytomyptera* Rondani from Myanmar, and three new species of *Rossimyiops* Mesnil from Thailand and Myanmar. Keys are provided to the known species of *Perumyia* and *Rossimyiops* and general aspects of the webspinner–tachinid association are discussed.

## Results

### Taxonomy

Tachinidae

Exoristinae, Goniini

***Embiophoneus*** **gen. nov.**

**LSID** urn:lsid:zoobank.org :act:866FB2D3-7B62-45C4-A9CE-873F42A50900

**Type species** *Embiophoneus rossi* **sp. nov.**, by present designation.

**Diagnosis** Small to medium-sized flies, mostly black in ground color. Compound eye bare. Two reclinate orbital setae (both sexes). Facial ridge slightly convex, with erect setae above vibrissa on lower 4/5. Lower facial margin not visible in lateral view in front of vibrissal insertion. Gena about 1/5 of compound eye height. Gena higher than width of parafacial measured at level of base of antenna. Postpedicel 4.0–4.5 times as long as pedicel. Four postsutural dorsocentral setae; one presutural and three postsutural intra-alar setae; first postsutural supra-alar seta stronger than other mesonotal setae, much longer than first postsutural intra-alar seta and longer than notopleural setae. Prosternum with a pair of fine setae laterally. Postpronotum with three setae arranged in a line. Katepimeron bare. Apical scutellar setae well developed. Anterior and posterior lappets of metathoracic spiracle about equal in size. Vein R_4 + 5_ with setae from base to junction with crossvein r-m. Wing cell r_4 + 5_ long petiolate. Preapical anterodorsal seta of fore tibia at least as long as preapical dorsal seta. Hind tibia with three dorsal preapical setae. Mid-dorsal depression of syntergite 1 + 2 extending on anterior half. Abdominal tergites 3 and 4 without median discal setae. Puparium light brown and reddish. Stigmatal plates located on prominent black protuberances.

**Etymology** The generic name is a composite word from Greek, with prefix “εμβιος”, i.e., “lively” (the prefix of the name Embioptera), and suffix “φονεύς”, i.e., “murderer”, referring to the parasitoid habits of the new taxon. The name is masculine.

**Distribution** Afrotropical: Ivory Coast, Liberia, Mozambique.

**Hosts** Embioptera.

***Embiophoneus rossi*** **sp. nov.**

**LSID** urn:lsid:zoobank.org :act: D69FEF7F-33B6–4905-BE41-E53033004A4F.

(Figs. 1 and 2)

**Fig. 1:**
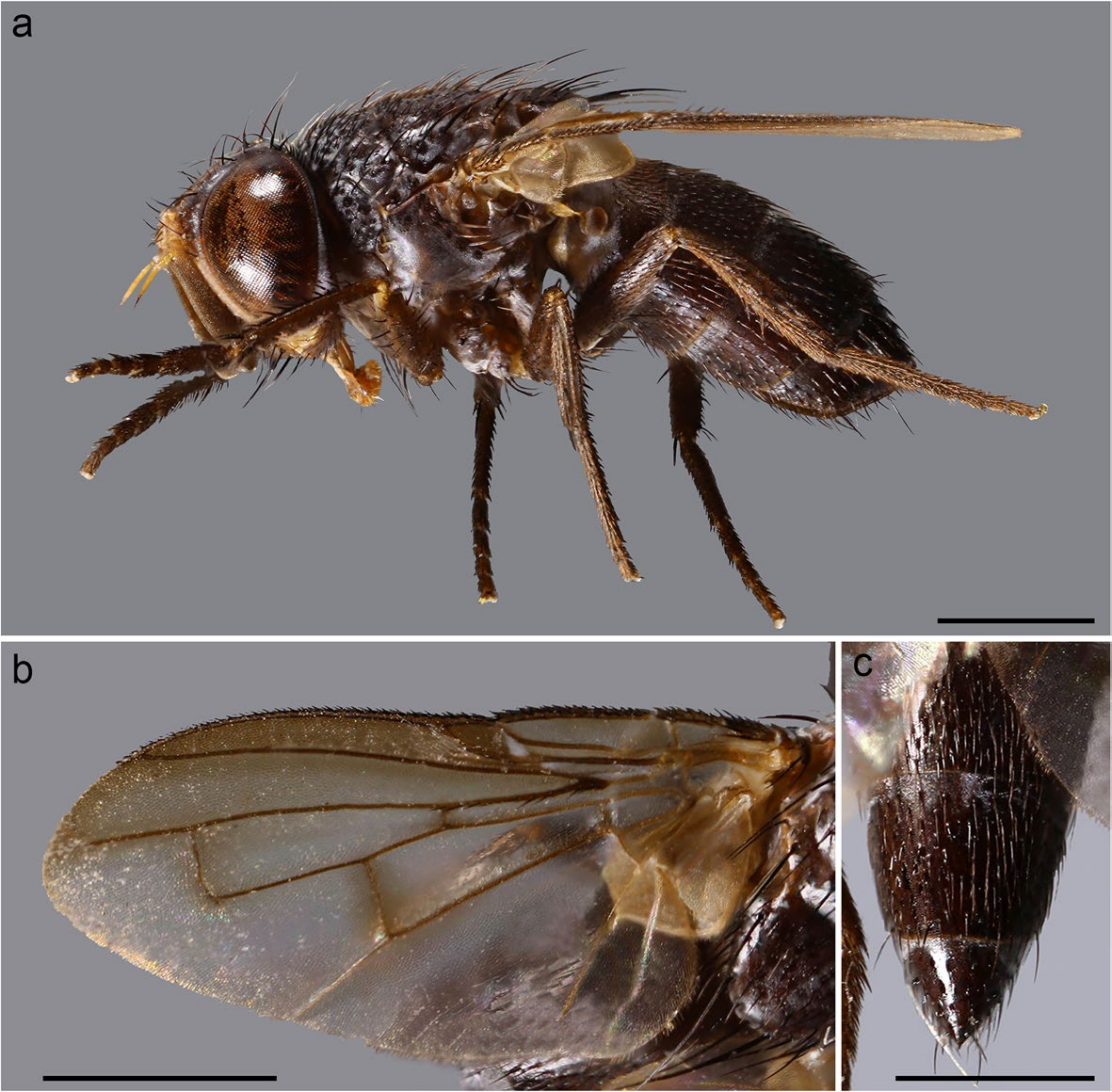
*Embiophoneus rossi* **gen.** et **sp. nov.**, male holotype. **a** habitus in lateral view. **b** wing. **c** abdomen in dorsal view. Scale bar 1 mm

**Fig. 2:**
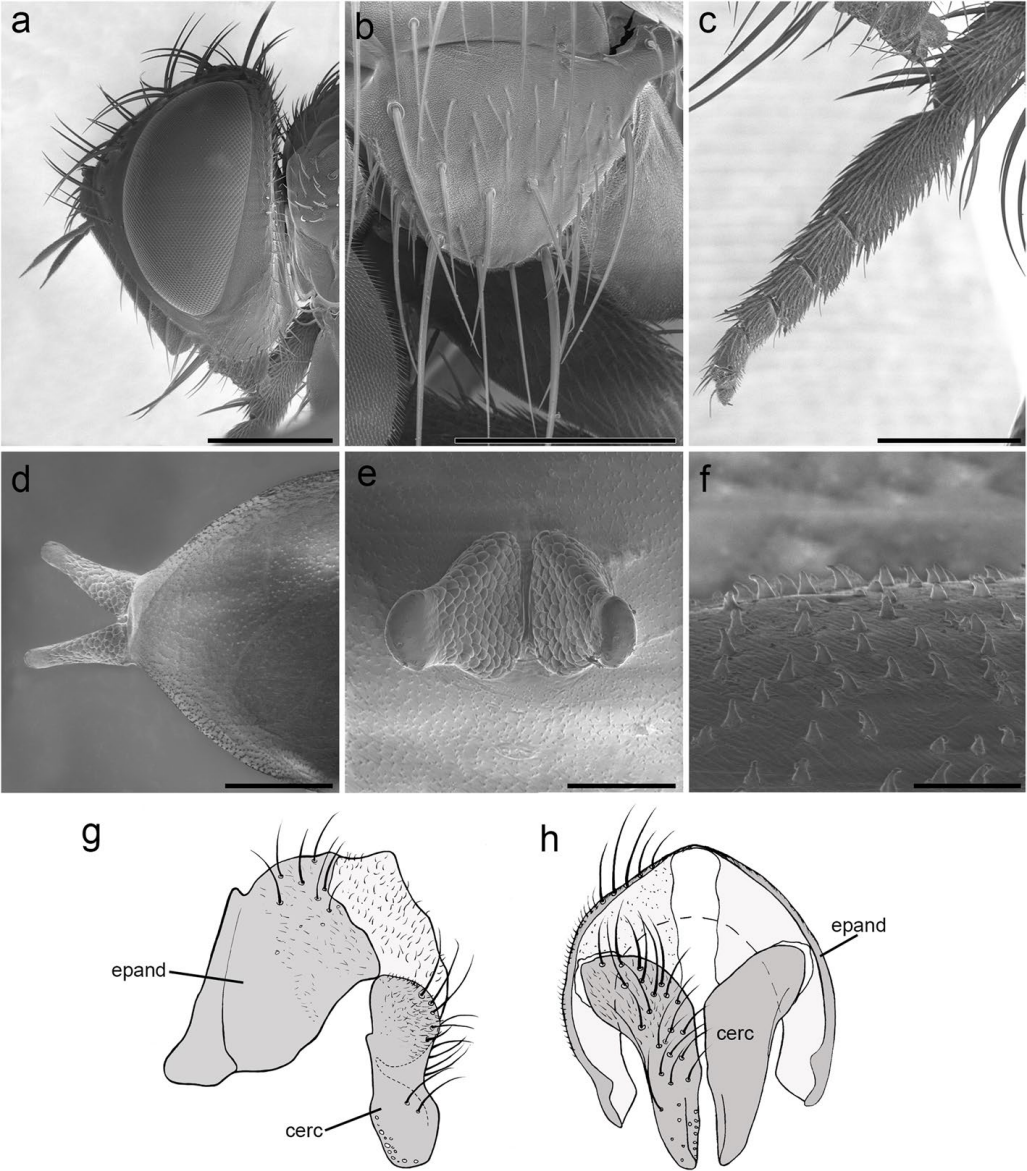
*Embiophoneus rossi* **gen.** et **sp. nov.**, male holotype. **a** head in lateral view at SEM. Scale bar 500 μm **b** scutellum in dorsal view at SEM. Scale bar 500 μm. **c** fore tarsus at SEM. Scale bar 500 μm. **d–f** Puparium at SEM: **d** posterior spiracles of puparium in dorsal view. Scale bar 500 μm. **e** posterior spiracles in posterior view. Scale bar 300 μm. **f** puparium surface and ornamentation. Scale bar 50 μm. **g–h** *E. rossi* **sp. nov.** male holotype, terminalia, **g** in lateral view, **h** in posterior view

**Diagnosis** Body length about 4–5 mm. Arista bare and thickened on proximal 2/3. Apical scutellar setae strong and crossed. Wing membrane with a light shadow on anterior part. Bend of vein M_1_ forming a nearly right angle. Bend of vein M_1_ without stub. Cell r_4 + 5_ with petiole about 1.3 times postangular section of vein M_1_. Abdomen black; tergites 3 and 4 with a narrow anterior band of pruinosity. Syntergite 1 + 2 without median marginal setae. Tergite 5 very short, about 0.6 times as long as tergite 4. Anterior tarsus enlarged. Epandrium short and convex.

**Description (male)** Body length: 4.6 mm. *Color* (Fig. 1)*.* Head brown. Scape and pedicel light brown. Postpedicel brown. Arista yellow. Palpus yellow. Thorax brown, mostly covered with whitish pruinosity except on four dark vittae. Upper and lower calypters yellowish. Wing hyaline with a light brown shadow on anterior part, along costa. Tegula and basicosta brown. Wing veins brown. Scutellum mainly dark brown, covered with pruinosity. Abdomen black; tergites 3 and 4 with a narrow anterior band of pruinosity. Femora and tibiae dark brown. *Head* (Fig. 2a). Frons at its narrowest point about 4/5 as wide as a compound eye width in dorsal view. Outer vertical seta present and well developed. Ocellar seta well developed and latero-proclinate. Frontal setae descending to level of arista insertion. Fronto-orbital plate more or less setulose. Two proclinate orbital setae. Parafacial bare below lowest frontal seta. Parafacial at its narrowest point approximately 2/5 of width of postpedicel at mid length. Parafacial measured ventrally at its narrowest point 1/4 of distance between inner margin of compound eye and antennal insertion. Vibrissa inserted at level of lower facial margin. Face and lower facial margin not visible in lateral view in front of vibrissal insertion. Genal dilation well developed. Ventral and dorsal part of occiput with a majority of black setae. Postpedicel 4.2 times as long as pedicel. Arista bare and thickened on proximal 2/3. Second aristomere 1.5 times as long as its diameter. Prementum stubby, 2 times as long as wide. Palpus apically enlarged. *Thorax.* Three presutural and three postsutural acrostichal setae; three presutural dorsocentral setae. Katepisternum with three setae. Scutellum with five pairs of marginal setae (basal, two laterals, subapical, apical); subapical scutellar setae well developed; apical scutellar setae strong and crossed; preapical scutellar setae absent (Fig. 2b). *Wing* (Fig. 1b). Second costal section (Cs_2_) setulose ventrally. Costal spine not differentiated from other costal setae. Veins R_1_ and M_4_ bare. Bend of vein M_1_ forming a nearly right angle. Bend of vein M_1_ without stub. Section between crossveins r-m and dm-m shorter than section between dm-m and postangular section of vein M_1_. Cell r_4 + 5_ with a petiole about 1.3 times postangular section of vein M_1_. Vein CuA + CuP not reaching wing margin. *Legs*. Legs stout. Fore coxa with anteroventral surface bare. Anterior tarsus enlarged (Fig. 2c). Fore tibia with preapical anterodorsal seta about same length of dorsal preapical seta. Mid tibia with one anterodorsal seta. Anterodorsal setae of hind tibia irregularly spaced and irregular in size. *Abdomen* (Fig. 1c). Tergites 1 + 2 and 3 without median marginal setae. Tergites 4 with a row of short erect marginal setae. Tergite 5 very short, about 0.6 times as long as tergite 4. *Male terminalia* (Fig. 2g, h). Epandrium short and convex. Cerci well developed, covered with setae; apical third of cerci separated and tips gently converging medially, in posterior view. Phallus, surstylus and hypandrial complex not examined, missing in the holotype. *Puparium*. Ground color from light brown to reddish, posterior spiracle black. Posterior spiracle horn-like (i.e., with a relatively large, sub-elliptical base, gently tapering distally with rounded apex) (Fig. 2d): lateral surface with a cobblestone-like microsculpture, posterior end smooth with several small, sub-elliptical openings (Fig. 2e). Surface evenly covered with minute, spine-like, protuberances (Fig. 2f).

**Distribution** Ivory Coast, Liberia, Mozambique.

**Hosts** Embioptera: unidentified species of *Parachirembia* Davis (Embiidae) (labelled with an unavailable species name by Ross) (Liberia); unidentified species of *Parachirembia* (Embiidae) (Ivory Coast); undescribed species (labelled with an unavailable genus name by Ross) (family not given, likely Embiidae [K.B.M, unpublished]) (Mozambique).

**Etymology** The species is dedicated to Edward S. Ross in recognition of his life-long contribution to our knowledge of Embioptera.

**Type material** Holotype ♂: HOLOTYPE ♂ / *Embiophoneus* / *rossi* sp. nov. / D. Badano et al. det. / 2021 // Host *Parachirem* / *bia liberica* Ross [unavailable species name] / Liberia: 10 mi. N. / Gbanka 25.XI. / 1966 E. S. Ross // Collection of the California Academy of Sciences, San Francisco, Calif. (Fig. S1 in Additional file 1) [CAS]. Paratype ♂: Host *Parachirem-* / *bia liberica* Ross [unavailable species name] / Liberia: 10 Mi. N. / Gbanka. 25.XI. / 1966 E. S. Ross // Collection of the California Academy of Sciences, San Francisco, Calif. Paratype ♂: Mozambique: 56 MI. / SW. Namacurra alt. / 100′ 12.VII.1972 E. S. Ross // Emerged from *Dicrocerembia* [unavailable genus name] / 16.XI.1972 at / San Francisco / Host collected at: // Collection of the California Academy of Sciences, San Francisco, Calif. // Goniini / det. D.M. Wood, 2012. Paratype ♀: Ivory Coast / Abidjan / Foret de Banco / IX-10-1958 // Ex. / *Parachirembia* / n. sp. // E. S. Ross / collector // Collection of the California Academy of Sciences, San Francisco, Calif. [All in CAS.]

**Remarks** *Embiophoneus rossi* likely belongs to the Eryciini + Goniini clade (Exoristinae) based on the combination of setulose prosternum, strong first postsutural supra-alar seta, convex facial ridge with a row of strong setae above vibrissa and cerci not fused medially. The dissected female had no eggs stored in uterus; thus, it is not possible to ascertain whether *Embiophoneus* is micro- (i.e., Goniini) or macro-ovolarviparous (i.e., Eryciini) [21]. However, *Embiophoneus* is likely a member of Goniini, with which it shares a short oviscapt [19]. Among Goniini, *Embiophoneus* is similar to the Palaeotropical genus *Prosopodopsis* Townsend, from which it is readily distinguishable by the long petiolate wing cell r_4 + 5_.

***Perumyia*** Arnaud, 1963

*Perumyia* Arnaud, 1963: 2. Type species: *Perumyia embiaphaga* Arnaud, 1963, by original designation.

**Diagnosis** (modified from Arnaud [19]) Small to medium-sized flies. Compound eye bare. Frons at its narrowest point 1.0–1.2 times as wide as compound eye width in dorsal view (both sexes). Two proclinate orbital setae (female). Ocellar seta well developed and reclinate. Parafacial bare below lowest frontal seta. Gena about 1/5 of compound eye height. Arista micropubescent, thickened nearly to tip. Prosternum setulose. Scutellum with three or four marginal setae. Wing hyaline. Cell r_4 + 5_ closed and long petiolate. Mid femur with two or more anterior setae. Mid tibia with two strong anterodorsal setae. Mid-dorsal depression of syntergite 1 + 2 extending to posterior margin. Tergite 3 and 4 without median discal setae.

**Distribution** Neotropical: Mexico (new record), Nicaragua (new record), Peru [19].

**Hosts** Embioptera.

***Perumyia embiaphaga*** Arnaud, 1963

*Perumyia embiaphaga* Arnaud, 1963: 4. Type locality: Pucallpa (Peru).

**Fig. 3:**
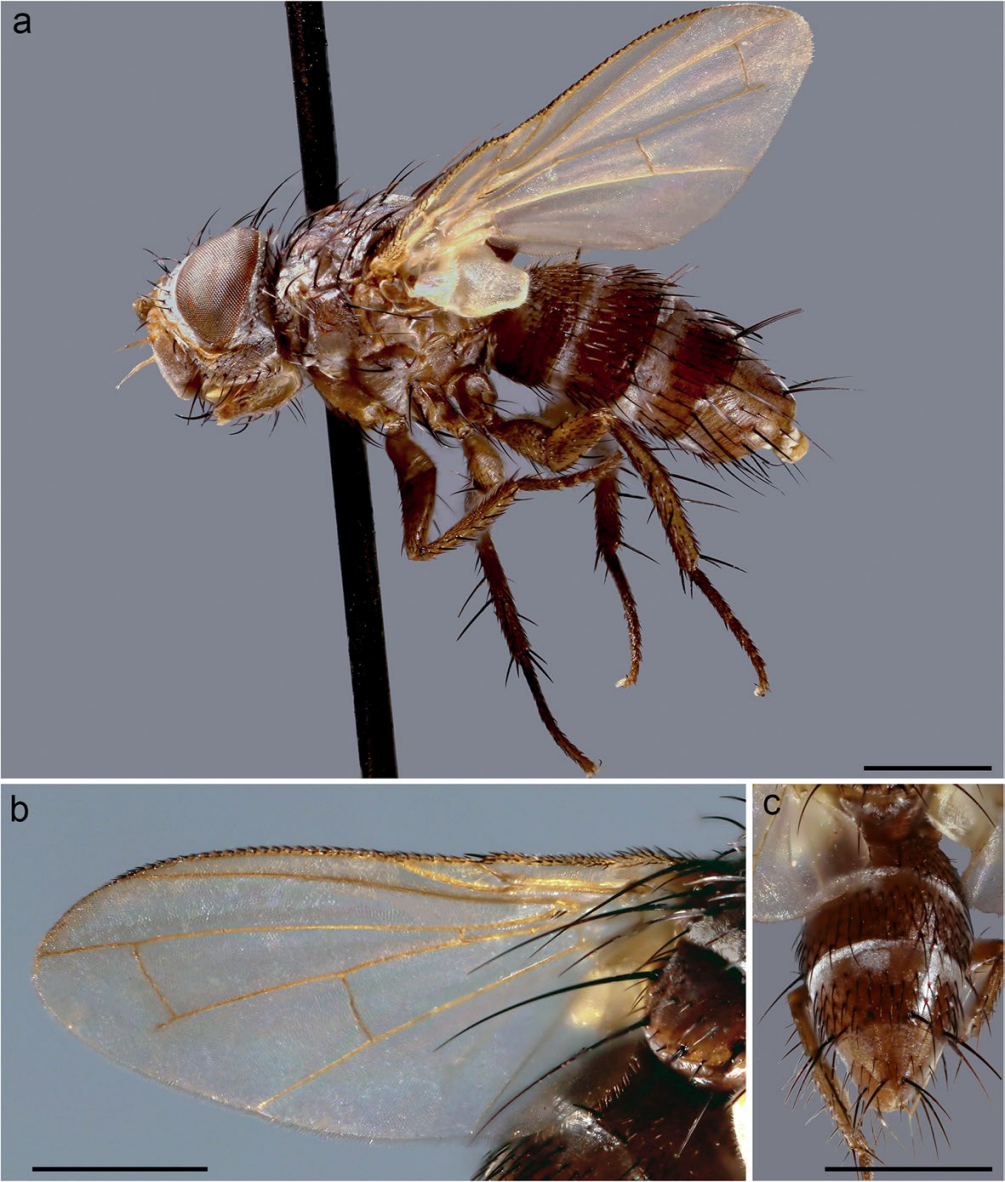
*Perumyia embiaphaga* Arnaud. **a** habitus in lateral view. **b** wing. **c** abdomen in dorsal view. Scale bar 1 mm

**Description** Body length: 5 mm. *Color* (Fig. 3). Head brownish covered with silver-grey pruinosity. Scape, pedicel and postpedicel yellowish. Palpus yellowish. Thorax reddish-brown in ground color. Presutural area with whitish pruinosity except on four dark vittae. Upper and lower calypters whitish. Tegula black and basicosta yellow. Wing veins light brown. Legs reddish-brown in ground color. Scutellum mainly brown with pruinosity. Abdomen mostly light brown and partly orange laterally, with a band of whitish pruinosity on posterior part of tergites. Tergites 3 and 4 with a band of pruinosity on anterior 1/3.

**Fig. 4:**
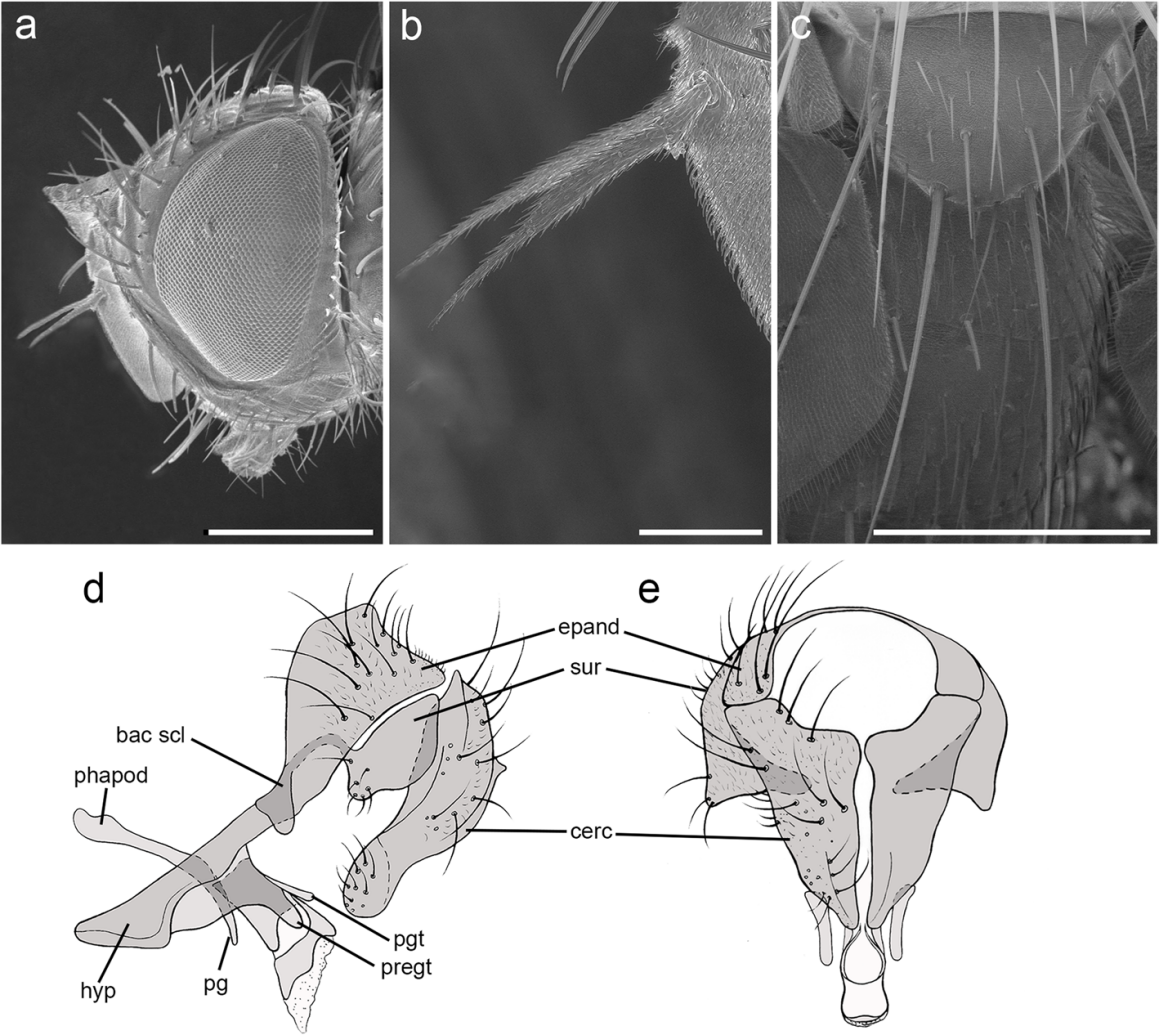
*Perumyia embiaphaga* Arnaud. **a** head in lateral view at SEM. **b** detail of arista in lateral view at SEM. **c** scutellum with hair-like setae in dorsal view at SEM. **d–e** male terminalia, **d** in lateral view. **e** in posterior view. Scale bar 500 μm

*Head* (Fig. 4a). Outer vertical seta present and well developed (both sexes). Frontal setae descending to level of arista insertion. Fronto-orbital plate with a row of reclinate or medioclinate setae and some hair-like setae. Parafacial at its narrowest point approximately 2/5 width of postpedicel at mid length. Parafacial measured ventrally at its narrowest point approximately 1/4 distance between inner margin of compound eye and antennal insertion. Facial ridge slightly convex and with erect setae above vibrissa on lower 3/5. Vibrissa inserted at level of lower facial margin. Face not visible in lateral view. Lower facial margin slightly visible in lateral view in front of vibrissal insertion. Genal dilation developed. Ventral and dorsal part of occiput with majority of white setae. Antenna longer than height of gena. Postpedicel 3 times as long as pedicel (Fig. 4a). Arista thickened on proximal 2/3 (Fig. 4b). First aristomere very short, shorter than wide. Second aristomere 2 times as long as its diameter. Palpus apically enlarged. *Thorax.* Scutum with three presutural and three postsutural acrostichal setae; three presutural and three postsutural dorsocentral setae; three postsutural intra-alar setae; first postsutural supra-alar seta longer than notopleural setae. Postpronotum with two setae. Katepisternum with two setae. Katepimeron bare. Scutellum with three pairs of marginal setae (basal, subapical, apical); apical scutellar setae hair-like and crossed (Fig. 4c); preapical scutellar setae absent; anterior and posterior lappets of metathoracic spiracle about equal in size. *Wing* (Fig. 3b). Costal spine as long as crossvein r-m. Vein R_1_ bare. Vein R_4 + 5_ with two setae at base. Bend of vein M_1_ with stub as long as crossvein r-m. Cell r_4 + 5_ with petiole 0.9 times as long as the postangular section of vein M_1_. Section of vein M_1_ between crossveins r-m and dm-m shorter than section between dm-m and bend of vein M_1_. *Legs*. Preapical anterodorsal seta of fore tibia longer than dorsal preapical seta. Hind tibia with three dorsal preapical setae. Preapical posteroventral seta of hind tibia shorter than preapical anteroventral seta. Anterodorsal setae of hind tibia irregular in size. *Abdomen* (Fig. 3c)*.* Tergites 3 with one pair of median marginal setae; tergite 4 with a complete row of marginal setae. Tergite 5 approximately as long as tergite 4. *Male terminalia* (Fig. 4 d, e)*.* Epandrium short and convex with well-developed anterior prolongation. Cerci not fused medially, more or less subparallel and distally rounded in lateral view, more or less sharpened in posterior view. Cercus with strong setae and covered by hair-like setae. Surstylus very short, not fused to epandrium, approximately 1/3 as long as cercus. Surstylus with short setae distally. Phallus short. Epiphallus absent. Medioventral sclerite of distiphallus absent; extension of dorsal sclerite of distiphallus not developed. Pregonite well developed, fused to hypandrium and lobe-like. Postgonite small, lobe-like and bare. Bacilliform sclerite very short, s shaped. Phallus apodeme and phallic guide well developed. Medial plate of hypandrium short and convex. Hypandrial arms firmly fused posteromedially, entirely encircling base of phallus. *Puparium*. Not preserved in examined specimens.

**Distribution** Peru.

**Hosts** Embioptera: unidentified species of *Clothoda* Enderlein (Clothodidae) (Peru), and *Archembia* Ross (Archembiidae) (Peru).

**Type material examined** One female paratype from Peru, Pucallpa, November 7, 1954, emerged from *Clothoda* sp. [19] [CAS; examined by P.C.].

**Other material examined** ♂: Peru: Cueva de la Paves. Nr Tingo Maria // Ex *Archembia* // Mat. In Culture VII-17-1964 // Collection of the California Academy of Sciences San Francisco, Calif [CAS].

**Remarks** The male shows all diagnostic characters of *Perumyia embiaphaga*, except for vein R_4 + 5_ bearing two setae at base instead of six, which likely represents intraspecific variability.

***Perumyia arnaudi*** **sp. nov.**

**LSID** urn:lsid:zoobank.org:act:4DB1244A-179C-4D28-85CA-FDE3188F7B2D.

**Diagnosis** Body length about 4–5 mm. Frons at its narrowest point as wide as compound eye in dorsal view (both sexes). Second aristomere 3.5–4.0 times as long as its diameter. Apical scutellar setae absent. Vein R_4 + 5_ with short setae from base approximately halfway to crossvein r-m. Bend of vein M_1_ with a stub longer than crossvein r-m. Abdomen brown and partly reddish laterally, tergites 3 and 4 with pruinosity on anterior 1/3.

**Fig. 5:**
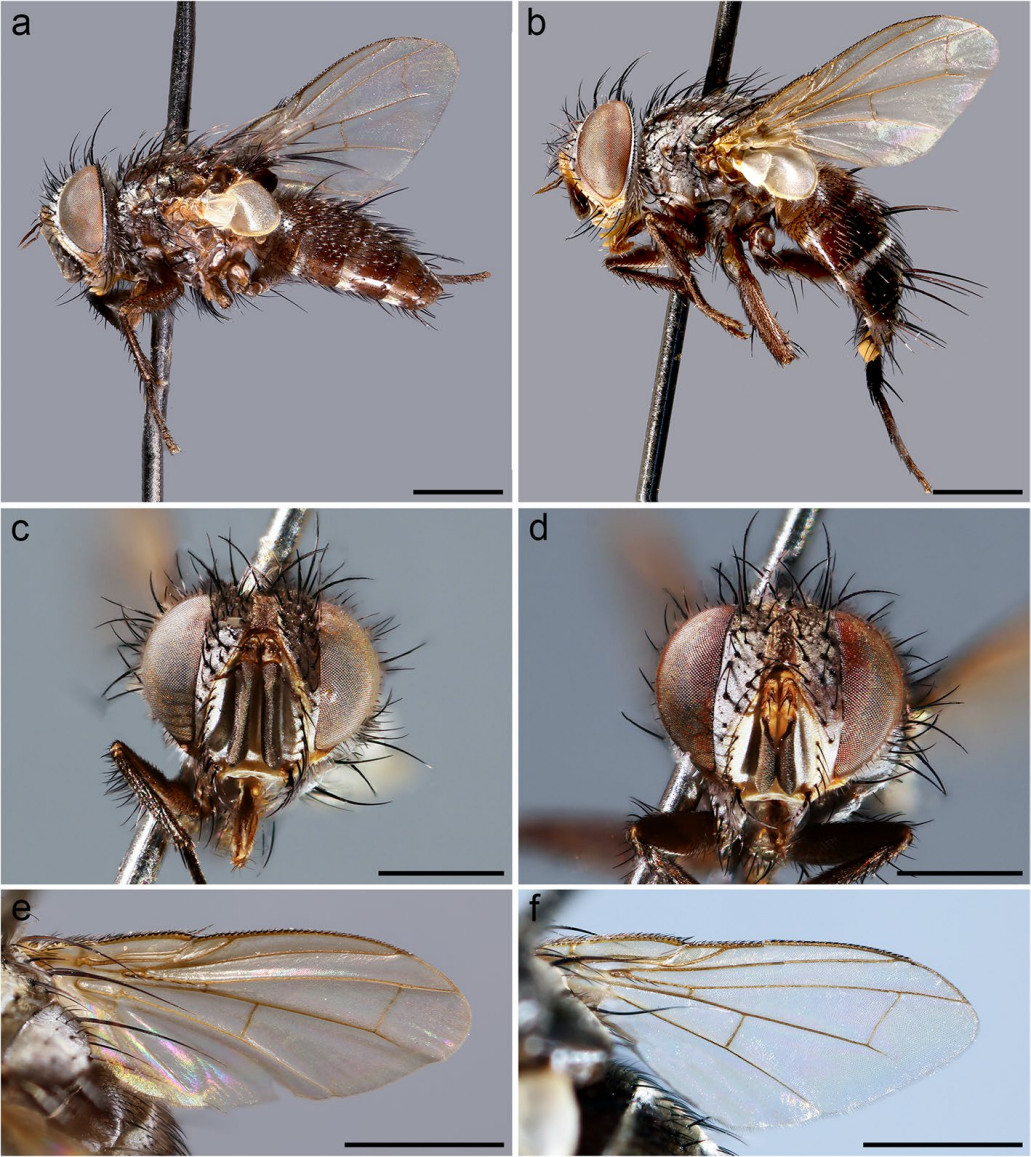
*Perumyia arnaudi* **sp. nov.**, **a** male paratype, habitus in lateral view. **b** female paratype, habitus in lateral view. **c** male paratype, head in frontal view. **d** female paratype, head in frontal view. **e** male paratype wing. **f** female paratype wing. Scale bar 1 mm

**Fig. 6:**
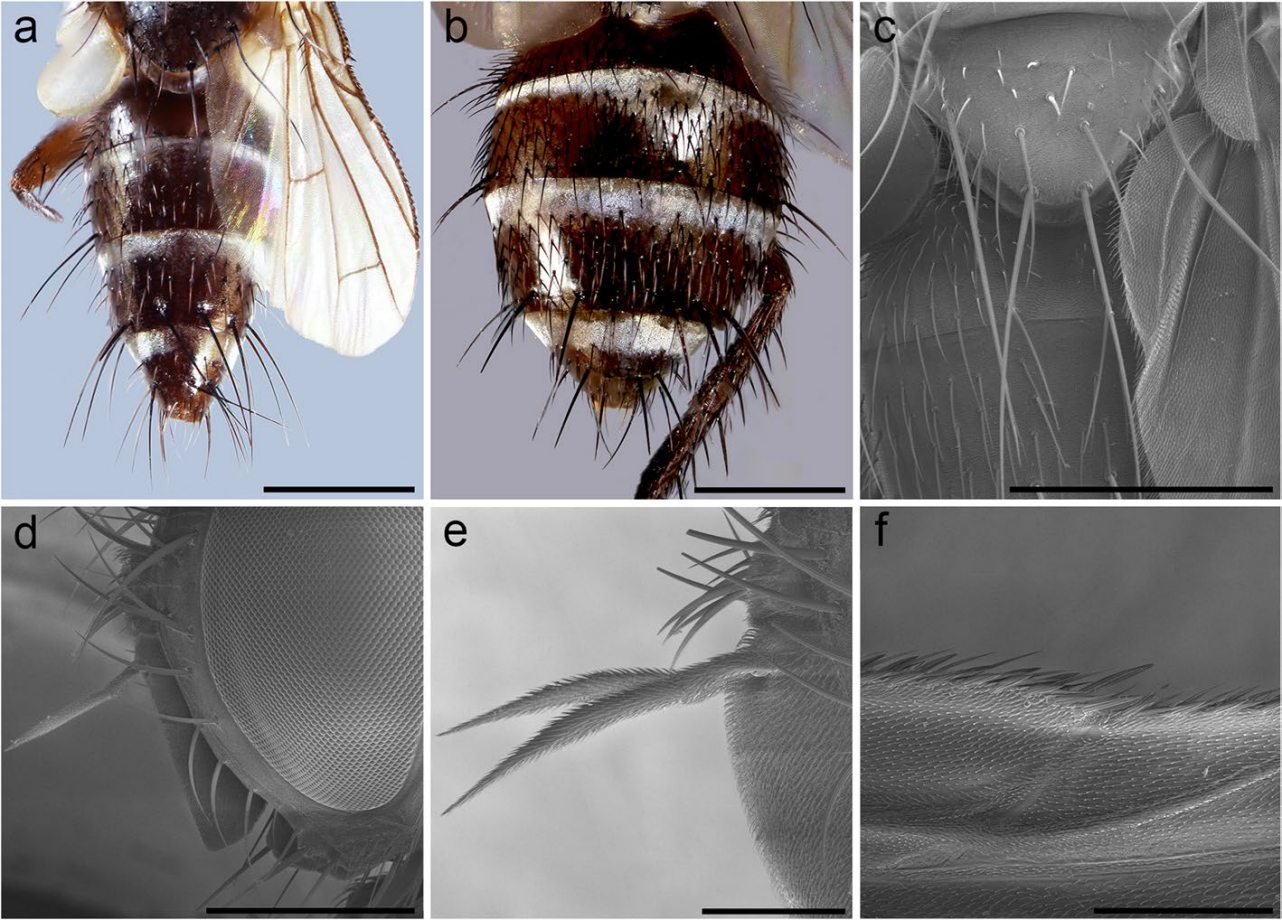
*Perumyia arnaudi* **sp. nov.**, **a** male paratype, abdomen in dorsal view. Scale bar 1 mm. **b** female paratype, abdomen in dorsal view. Scale bar 1 mm. **c** scutellum in dorsal view at SEM. Scale bar 500 μm. **d** male paratype, head in lateral view at SEM. Scale bar 500 μm. **e** arista detail in lateral view at SEM. Scale bar 200 μm. **f** detail of wing costal spine at SEM. Scale bar 200 μm

**Description (male, differences with females are given)** Body length: 4.7 mm. *Color* (Fig. 5 a, b)*.* Head dark brown covered with silvery-grey pruinosity. Scape and pedicel brown (male) or yellowish (female). Postpedicel mostly dark brown shading into yellowish at junction with pedicel. Palpus yellowish. Thorax dark brown. Presutural area with whitish pruinosity except on four dark vittae. Upper and lower calypters whitish. Wing veins brown or yellowish. Tegula black and basicosta yellow. Legs brown in ground color. Scutellum mainly brown with pruinosity. Abdomen brown and partly reddish laterally. Tergites 3 and 4 with pruinosity on anterior 1/3. Terminalia brown. *Head* (Fig. 5c, d). Frons at its narrowest point as wide as compound eye in dorsal view (both sexes). Outer vertical seta present and well developed (both sexes). Frontal setae descending slightly below level of arista insertion. Fronto-orbital plate with a row of reclinate or medioclinate setae and some air-like setae lateral to row of frontal setae. Parafacial at its narrowest point approximately 2/5 width of postpedicel at mid length. Parafacial measured ventrally at its narrowest point 1/4 distance between inner margin of compound eye and antennal insertion. Facial ridge slightly convex and with erect setae above vibrissa on lower 3/5. Vibrissa inserted at level of lower facial margin. Face not visible in lateral view. Lower facial margin slightly visible in lateral view in front of vibrissal insertion. Genal dilation developed. Ventral and dorsal part of occiput with a majority of white setae. Antenna longer than height of gena. Postpedicel 5 times as long as pedicel. Arista thickened on proximal 4/5. First aristomere very short, shorter than wide. Second aristomere 3.5–4.0 times as long as its diameter (Fig. 6d, e). Palpus apically enlarged. *Thorax.* Scutum with three presutural and three postsutural acrostichal setae; three presutural and three postsutural dorsocentral setae; three postsutural intra-alar setae; first postsutural supra-alar seta longer than notopleural setae. Postpronotum with two setae. Katepisternum with two setae. Katepimeron bare. Scutellum with three pairs of marginal setae (basal, lateral, subapical) (Fig. 6c); apical and preapical scutellar setae absent. Anterior and posterior lappets of metathoracic spiracle about equal in size. *Wing* (Fig. 5e, f). Costal spine as long as crossvein r-m (Fig. 6f). Vein R_1_ bare. Vein R_4 + 5_ with short setae from base approximately halfway to crossvein r-m. Bend of vein M_1_ with stub longer than crossvein r-m. Cell r_4 + 5_ with petiole 1.1 times as long as postangular section of vein M_1_. Section of vein M_1_ between crossveins r-m and dm-m shorter than section between dm-m and bend of vein M_1_. *Legs*. Preapical anterodorsal seta of fore tibia about the same length as dorsal preapical seta. Hind tibia with three dorsal preapical setae. Preapical posteroventral seta of hind tibia shorter than preapical anteroventral seta. Anterodorsal setae of hind tibia irregular in size. *Abdomen* (Fig. 6a, b)*.* Tergite 3 with one pair of median marginal setae; tergite 4 with a complete row of marginal setae. Tergite 5 shorter than tergite 4. *Male terminalia*. As for genus. *Puparium*. Not preserved in examined specimens.

**Distribution** Mexico, Nicaragua.

**Hosts** Embioptera: unidentified species of *Mesembia* Ross (Anisembiidae) (Mexico) and *Neorhagadochir* Ross (subgenus *Drepanembia* Ross) (Scelembiidae) (Nicaragua).

**Etymology** The species is dedicated to the entomologist Paul H. Arnaud in recognition of his contribution to our knowledge of Tachinidae.

**Type material** Holotype ♂: HOLOTYPUS ♂ / *Perumyia* / *arnaudi* sp. nov. / Badano et al. det. 2021 // Nicaragua: 31 mi. / NW Esteli, 2000 ft. / 29-Nov-1976 / Host: *Drepanembia* // Fly matured 18-Nov- / 1976 / Ex *Drepanembia*, / Edward S. Ross. **//** Collection of the California Academy of Sciences, San Francisco, Calif. (Fig. S1 in Additional file 1) [CAS]. Paratype ♂: Mexico: Chiapas. / 7 mi. E. of San / Cristobal las Casas, / 7200 ft. Host *Mes*- // *embia* n. sp. / Fly matured 19-Nov- / 1976 / Ex, *Mesembia* n. sp. / Edward S. Ross // Collection of the California Academy of Sciences, San Francisco, Calif. Paratype ♀: Nicaragua: 31 mi. / NW Esteli, 2000 ft. / 29-Nov-1976 / Host: *Drepanembia* // Fly matured 24-May- / 1984 / Ex *Drepanembia*, Edward S. Ross // Collection of the California Academy of Sciences, San Francisco, Calif. // Goniini / *Peruana* [sic] / *embiaphaga* / det: D.M. Wood, 2012. [All in CAS.]

### **Identification key to*Perumyia*species**

1. Frons at narrowest point as wide as compound eye in dorsal view. Postpedicel 5 times as long as pedicel. Vein R_4 + 5_ with short setae from base approximately halfway to crossvein r-m (Fig. 5e, f). Apical scutellar setae absent ..........................*Perumyia arnaudi* sp. nov.

- Frons at narrowest point about 1.0–1.2 times as wide as compound eye in dorsal view. Postpedicel 3 times or less as long as pedicel. Vein R_4 + 5_ with 2–6 short setae at base. Apical scutellar setae present ...........................................*Perumyia embiaphaga* Arnaud

**Remarks** The Neotropical genus *Perumyia* was erected by Arnaud [19] for *P*. *embiaphaga*. This species was described on seven specimens emerged from a Peruvian species of *Clothoda* [19]. The puparia of the examined specimens of *Perumyia* were enveloped in embiid silk, i.e., not enclosed within the remains of the host, suggesting that the larva leaves the host body before pupating [19]. The affinities of *Perumyia* are unclear but it appears it may form a monophylum with the New World goniine genus *Distichona* Wulp. *Perumyia* and *Distichona* share the characteristic reclinate ocellar setae of the *Gonia* Meigen group of genera, as well as the following character states: narrow parafacial (parafacial can be bare or with setae in *Distichona*), strong rows of both reclinate orbital setae and proclinate setae on facial ridge. *Distichona* includes 8 species [16]—note that 11 species is given in Wood & Zumbado [22]—ranging from southern Canada to Peru and differs from *Perumyia* by the non-petiolate wing cell r_4 + 5_, likely a plesiomorphic condition. The hosts of *Distichona* remain unknown, despite a recent report that suggested otherwise. A Mexican study published in English by Salas-Araiza [23] and in Spanish by Salas-Araiza & González-Márquez [24] reported *Distichona auriceps* Coquillett as a newly recorded parasitoid of the fall armyworm, *Spodoptera frugiperda* (J.E. Smith) (Lepidoptera: Noctuidae). As pointed out by O’Hara & Cerretti [25], this record was based on a misidentification; the tachinid identified as *D. auriceps* in Fig. 1 in Salas-Araiza & González-Márquez [24: 291] is *Archytas* sp. (Tachininae, Tachinini). [Similarly, another tachinid reared during the same study and identified in Fig. 2 as *Hypovoria discalis* (Brooks) (Dexiinae, Voriini) is *Winthemia* sp. (Exoristinae, Winthemiini) [25].

Tachininae, Graphogastrini

***Phytomyptera*** Rondani, 1845

*Phytomyptera* Rondani, 1845: 32, 33. Type species: *Phytomyptera nitidiventris* Rondani, 1845 [= *Tachina nigrina* Meigen, 1824], by monotypy.

**Diagnosis** Small to medium-sized flies, body length 2–5 mm. Frons of equal width or slightly wider than compound eye width in dorsal view (both sexes). Arista thickened at least on basal 2/3. Second aristomere 3–10 times as long as wide. Upper part of head with several rows of black setae behind postocular row (occipital area and genal dilation with only black setae). Prosternum setulose. Three postsutural intra-alar setae. Proepimeral seta curved downward. Scutellum with strong, convergent, subparallel or slightly diverging, subapical setae, apical setae present but very small. Vein R_4 + 5_ with a single large seta at base. Vein M_1_ with bend evenly curved or with apical section obliterated. Cell r_4 + 5_ not petiolate.

**Distribution** All biogeographic regions except Australasia/Oceania.

**Hosts** Lepidoptera Apodytrisia (several families). Embioptera (new record).

**Included species** See O’Hara et al. [16].

***Phytomyptera woodi*** **sp. nov.**

**LSID** urn:lsid:zoobank.org:act: F8221FBD-4C00-44EA-94D6-6A38367AF243.

**Diagnosis** Body length about 4 mm. Frons at its narrowest point approximately 1.2 times as wide as compound eye in dorsal view. Fronto-orbital plate with a few hair-like setae. Gena about 1/6 of compound eye height. Pedicel and postpedicel brown. Postpedicel large. Second aristomere approximately 7 times as long as its diameter. Palpus dark brown. Proepimeral seta strong and curved downward. Wing hyaline. Costal spine as long as crossvein r-m. Crossvein dm-m present. Vein M_1_ with a distinct bend and reaching wing margin. Cell r_4 + 5_ open. Distiphallus with a pair of narrow, membranous, lobe-like, lateroventral projections, covered with scale-like spinules. Medioventral sclerite of distiphallus (mesohypophallus of Salzer [26], see also Andersen [27] for *Phytomyptera*) well developed.

**Fig. 7:**
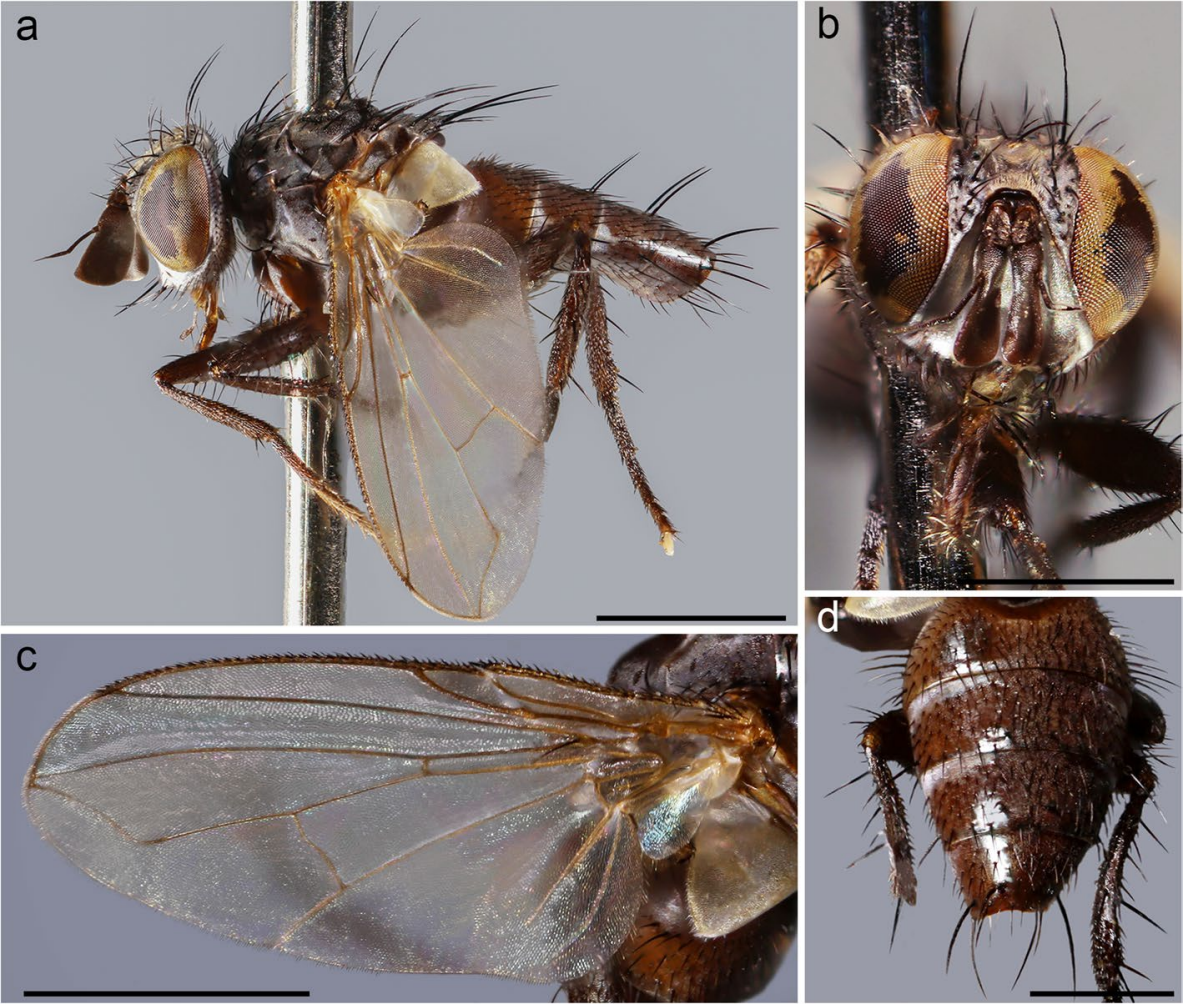
*Phytomyptera woodi* **sp. nov.**, male holotype. **a** habitus in lateral view. **b** head in frontal view. **c** wing. **d** abdomen in dorsal view. Scale bar 1 mm

**Fig. 8:**
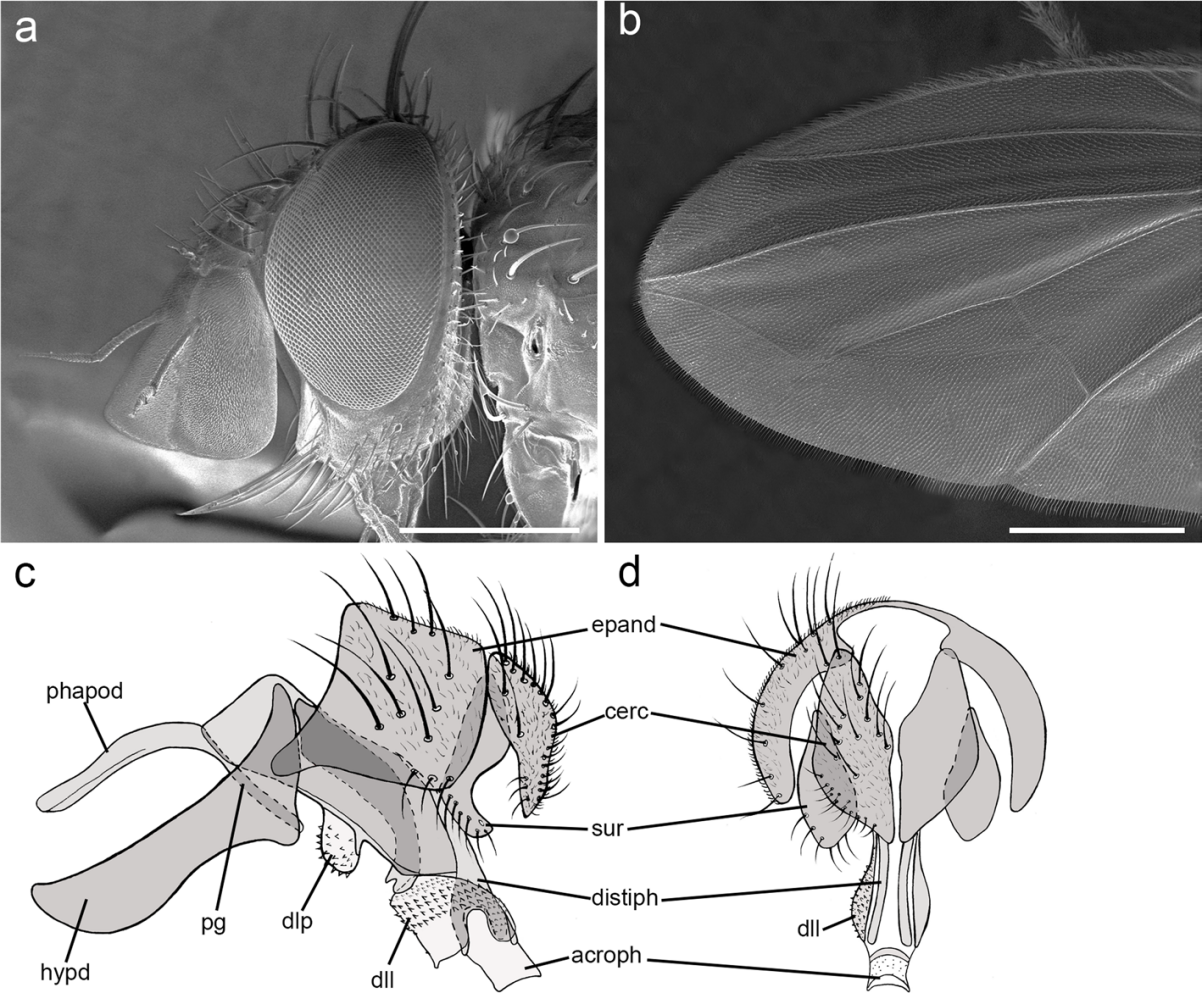
*Phytomyptera woodi* **sp. nov.**, male holotype. **a** head in lateral view at SEM. **b** wing apex at SEM. **c–d** male terminalia, **c** in lateral view, **d** in posterior view. Scale bar 500 μm

**Description (male)** Body Length: 4 mm. *Color* (Fig. 7)*.* Head black in ground color, covered with whitish pruinosity. Pedicel and postpedicel brown. Palpus dark brown. Thorax black. Presutural area with whitish pruinosity except on three dark vittae. Upper and lower calypter white. Scutellum black. Wing hyaline. Tegula dark brown and basicosta light brown. Wing veins dark brown. Legs and abdomen black. Tergites 3 and 4 each with a narrow anterior band of pruinosity interrupted along midline. Terminalia black. *Head* (Fig. 7b). Compound eye bare. Frons at its narrowest point about 1.2 times as wide as compound eye in dorsal view. Ocellar setae well developed and proclinate. Frontal setae descending to lower margin of pedicel. Fronto-orbital plate with a few hair-like setae. Two reclinate orbital setae. Parafacial bare below lowest frontal seta. Parafacial at its narrowest point 1/10 as wide as postpedicel at mid length. Facial ridge concave with erect setae above vibrissa on lower 1/8. Vibrissa inserted at level of lower facial margin. Face and lower facial margin not visible in lateral view in front of vibrissal insertion. Genal height 1/6 of compound eye height. Genal dilation well developed. Occiput with black hair-like setae. Postpedicel approximately 3 times as long as pedicel. Postpedicel axe head-shaped in lateral view wide: at its widest, distal point 0.65 times its length. First aristomere very short, no longer than wide. Second aristomere approximately 7 times as long as its diameter (Fig. 8a)*.* Prementum 5 times as long as wide. Palpus apically enlarged. *Thorax*. Scutum with two presutural acrostichal setae; two presutural and two postsutural dorsocentral setae; first postsutural supra-alar seta shorter than notopleural setae. Proepimeral seta strong and curved downward. Postpronotum with two setae. Katepisternum with three setae. Scutellum with three pairs of strong marginal setae (basal, lateral, subapical), apical scutellar setae thin and crossed; preapical scutellar setae straight and erect. Anterior and posterior lappets of metathoracic spiracle about equal in size. *Wing* (Fig. 7c). Second costal section (CS_2_) setulose ventrally. Costal spine as long as crossvein r-m. Vein R_1_ bare. Vein M_4_ bare. Crossvein dm-m present. Vein M_1_ with a distinct bend and reaching wing margin. Bend of vein M_1_ forming an obtuse angle. Vein M_1_ without stub. Section between crossvein r-m and dm-m approximately as long as section between dm-m and bend of vein M_1_. Cell r_4 + 5_ open (Fig. 8b). *Legs.* Preapical anterodorsal seta of fore tibia about the same length of dorsal preapical seta. Mid tibia with one anterodorsal seta. Hind tibia with two dorsal preapical setae. Preapical posteroventral seta of hind tibia shorter than preapical anteroventral seta. Anterodorsal setae of hind tibia unarranged and irregular in size. Posterodorsal margin of coxa bare. *Abdomen* (Fig. 7d). Mid-dorsal depression of syntergite 1 + 2 extending on anterior half. Syntergite 1 + 2 without median marginal setae. Both tergites 3 and 4 with one pair of median marginal setae; both without median discal setae. Tergite 5 approximately the same length of tergite 4. *Terminalia* (Fig. 8c, d). Epandrium short and convex, with well-developed anterior prolongation. Cerci not fused medially and more or less subparallel, apically pointed and curved anteriorly in lateral view. Cercus with strong erect setae on dorsal surface. Surstylus not fused to epandrium, approximately as long as cercus. Surstylus bent posteriorly in lateral view. Surstylus with strong setae on distal surface. Phallus stout. Epiphallus absent. Basal extensions of basiphallus not developed. Medioventral sclerite of distiphallus well developed. Distiphallus with a pair of narrow, membranous, lobe-like, lateroventral projections, covered with scale-like spinules (pleurohypophallus of Andersen [27]); extensions of dorsal sclerite of distiphallus (dorsal plate of Andersen [27]) wrench-like distally; lateroventral lobes of distiphallus well developed and covered with four rows of scale-like spinules (Fig. 8c). Acrophallus cylindrical, faintly sclerotized. Pregonite broad and scimitar shaped. Postgonite not developed. Bacilliform sclerite not differentiated. Phallus apodeme robust with a well-developed and long phallic guide. Hypandrium well developed, medial plate concave; hypandrial arms not fused. *Puparium*. Not preserved in examined specimen.

**Distribution** Myanmar.

**Hosts** Embioptera: undescribed species (labelled with an unavailable genus name by Ross) (family not given) (Myanmar).

**Etymology** The species is dedicated to the entomologist and eminent dipterist Donald Montgomery (Monty) Wood for his outstanding contribution to our knowledge of Tachinidae.

**Type material** Holotype ♂: HOLOTYPUS ♂ / *Phytomyptera* / *woodi* sp. nov. / Badano et al. det. 2021 // Burma: Maymyo, / 3538′, emerged 31- / Jan-1979, died 3- // Feb 1979, E. S. Ross, / collected in native / forest. Host is prob. / *Heoembia* [unavailable genus name] n. sp. // Collection of the California Academy of Sciences, San Francisco, Calif. // *Phytomyptera* sp. / det: D. M. Wood, 2012 [CAS] (Fig. S1 in Additional file 1).

**Remarks** *Phytomyptera* is a large and widespread genus of tachinines with 61 described species [16]. However, the actual diversity of this genus is much higher, given the large number of undescribed species preserved in museum collections (P.C., unpublished) and the lack of taxonomic revisions. Hosts are known only for a handful of species and are all microlepidopterans [27–29]. Despite the difference in host preference, morphological evidence supports *P. woodi* **sp. nov.** as a member of *Phytomyptera*. The monophyly of *Phytomyptera* is well supported by both molecular and morphological evidence [30]. The relationship of *P. woodi* **sp. nov.** to other species of *Phytomyptera* is still unclear. *Phytomyptera woodi* is readily distinguishable from the only other Oriental species, *P. minuta* (Townsend), as follows: wing vein M_1_ reaching wing margin (M_1_ not reaching wing margin in *P. minuta*), crossvein dm-m present (absent in *P. minuta*) and black palpus (yellow in *P. minuta*).

Tachininae, Minthoini

***Rossimyiops*** Mesnil, 1953

*Rossimyiops* Mesnil, 1953: 145. Type species: *Rossimyiops whiteheadi* Mesnil, 1953, by monotypy.

**Diagnosis** (modified from Cerretti et al. [20]) Small to medium-sized flies, body length varying from 2 to 6 mm. Compound eye bare. Male frons extremely narrow, and frontal vitta concealed by medial margin of fronto-orbital plate. Frons larger in female. Inner vertical setae parallel or crossed (only in Oriental species). Two or more proclinate orbital setae (female). Occiput with black setae only. Arista bare, thickened on proximal 1/5–1/2. Anterior and posterior lappets of metathoracic spiracle about equal in size. Apical scutellar setae crossed and horizontal or absent. Posterodorsal margin of hind coxa bare or with one strong seta (only in Oriental species). Mid-dorsal depression on abdominal syntergite 1 + 2 not extended posteriorly to posterior margin of that segment. Marginal setae on tergites 3, 4 and 5 “shifted” anteriorly into subdiscal position. Dorsolateral lobes of distiphallus well developed and “shifted” anteriorly. Surstylus distally bent posteriorly.

**Distribution** Palaearctic: Southeastern Europe [20, 31], Egypt (Sinai) [20, 32], Iran [33, 34], Iraq, Israel, Tunisia [20, 32, 35], Turkmenistan [36]. Afrotropical: Ethiopia 37], Namibia [20], Nigeria [37], South Africa [37–40]. Oriental: Myanmar (new record), Thailand (new record).

**Hosts** Embioptera.

Included species:

***Rossimyiops achilleae*** (Kugler, 1972)

*Mesnilomyia achilleae* Kugler, 1972: 107. Type locality: 'Arad (Israel).

**References** Cerretti et al. [20] [taxonomic review]

**Distribution** Egypt (Sinai), Israel.

**Hosts** Unknown.

***Rossimyiops aeratus*** **sp. nov.**

**LSID** urn:lsid:zoobank.org:act: A19CB1AF-2FD6-41A0-9E63-44242320F77A.

**Diagnosis** Body length: 3–4 mm. Inner vertical setae crossed. Ocellar setae well developed. Gena about 1/10 of compound eye height. Postpedicel 2 times as long as pedicel. Prosternum bare. Wing mostly hyaline, slightly smoky anterodistally. Vein R_4 + 5_ bare. Cell r_4 + 5_ closed at wing margin. Posterior margin of hind coxa with 1 strong seta. Abdomen dark brown without pruinosity. Abdominal discal setae absent. Male: epandrium and surstyli not fused.

**Fig. 9:**
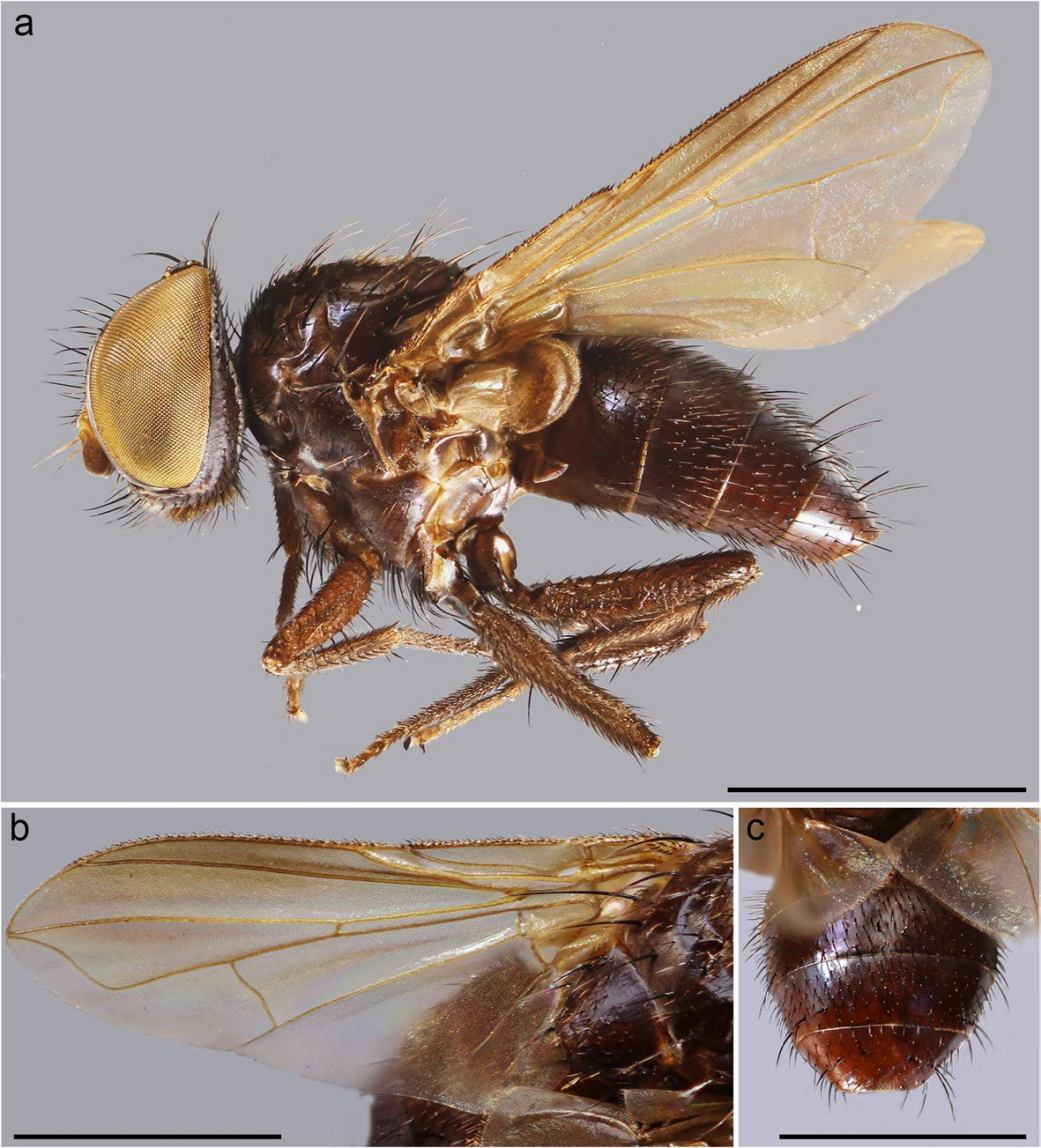
*Rossimyiops aeratus* **sp. nov.**, male holotype. **a** habitus in lateral view. **b** wing. **c** abdomen in dorsal view. Scale bar 1 mm

**Fig. 10:**
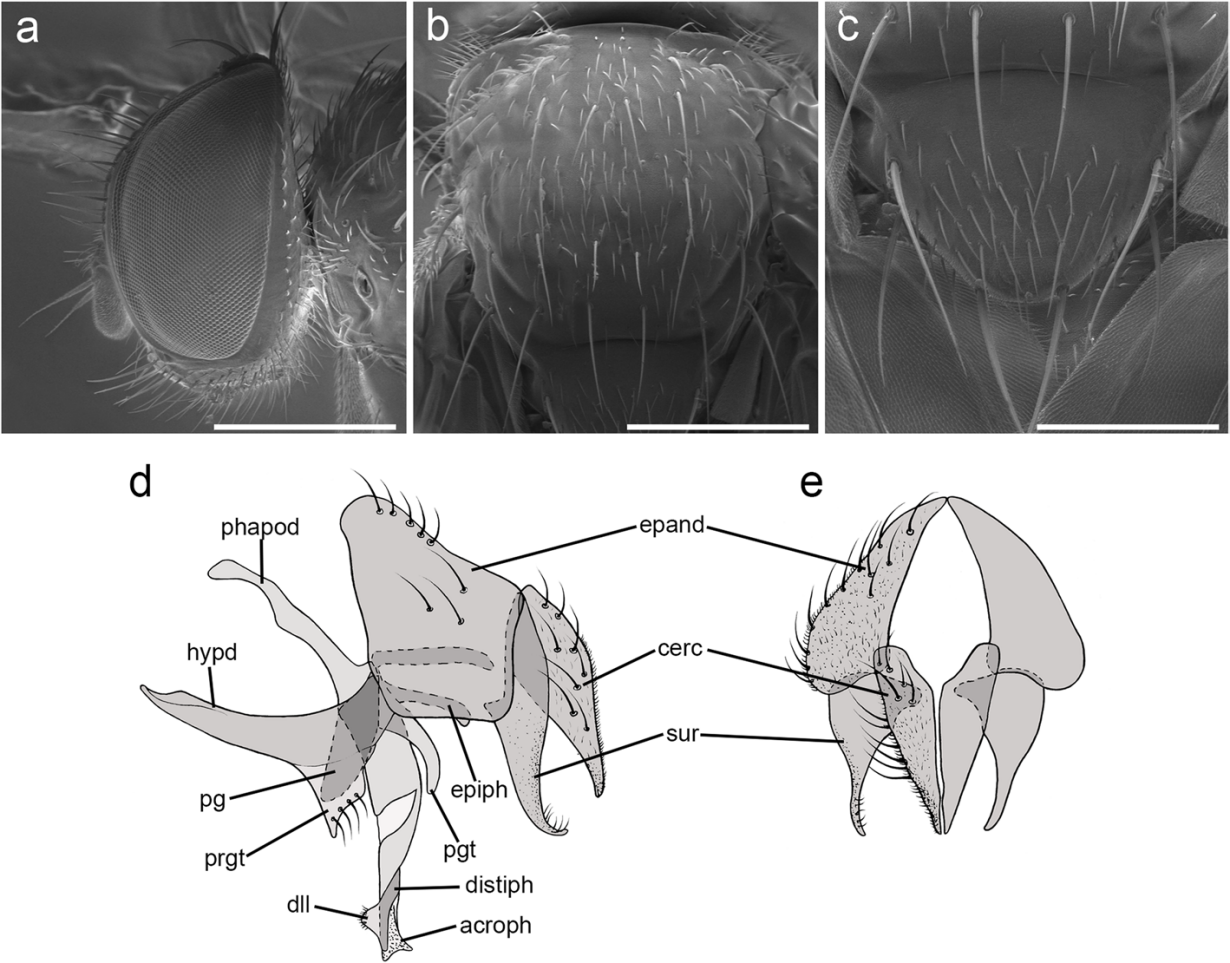
*Rossimyiops aeratus* **sp. nov.**, male holotype. **a** head detail in lateral view at SEM. **b** thorax in dorsal view at SEM. **c** scutellum in dorsal view at SEM. **d–e** male terminalia, **d** in lateral view, **e** in posterior view. Scale bar 500 μm

**Description (male)** Body length: 3.6 mm. *Color* (Fig. 9). Head black in ground color, covered with pruinosity. Scape and pedicel reddish-yellow. Postpedicel reddish-yellow proximally, shading into light brown distally. Palpus yellowish. Thorax dark brown. Presutural area without pruinosity and not showing longitudinal dark vittae. Upper and lower calypter brown with barely visible bronze reflections. Wing mostly hyaline, slightly darkened anterodistally (Fig. 9b). Tegula dark brown, basicosta light brown. Wing veins light brown to yellowish. Scutellum brown. Legs brown. Abdomen black, without pruinosity (Fig. 9c). Terminalia brown. *Head* (Fig. 10a). Frons at its narrowest point about 1/10 as wide as compound eye in dorsal view. Outer vertical seta not distinguishable from the rest of postocular setae. Inner vertical setae well developed and crossed. Ocellar seta well developed and proclinate. Frontal setae descending to upper margin of pedicel. Fronto-orbital plate bare. Proclinate and reclinate orbital setae absent. Parafacial at its narrowest point approximately 3/5–4/5 of width of postpedicel at mid length. Parafacial measured ventrally at its narrowest point 2/3 of minimum distance between inner margin of compound eye and antennal insertion. Parafacial bare below lowest frontal seta. Facial ridge slightly convex with setae above vibrissa on lower 1/5. Lower facial margin slightly visible in lateral view in front of vibrissal insertion. Gena about 1/10 of compound eye height. Genal dilation well developed. Postpedicel 2 times as long as length of pedicel. Arista bare and thickened on proximal 1/2. Prementum stubby, 1.8 times as long as wide. Palpus apically enlarged. *Thorax* (Fig. 10b). Scutum with one presutural acrostichal seta; two presutural and two postsutural dorsocentral setae; two postsutural intra-alar setae separated by a distance about equal to distance between first seta and suture; first postsutural supra-alar seta longer than notopleural setae. Prosternum bare. Posterior proepimeral seta upwardly curved. Postpronotum with two setae. Katepisternum with two setae. Scutellum with three pairs of marginal setae (basal, subapical, apical) (Fig. 10c); apical scutellar setae crossed; subapical scutellar setae shorter than apical setae; lateral scutellar setae absent. *Wing* (Fig. 9b). Costal spine not distinguishable from other costal setae. Veins R_1_ and R_4 + 5_ bare. Bend of vein M_1_ forming an obtuse angle. Section of vein M_1_ between crossveins r-m and dm-m approximately as long as section between dm-m and bend of vein M_1_. Cell r_4 + 5_ closed at wing margin. *Legs.* Preapical anterodorsal seta of fore tibia visibly longer than dorsal preapical seta. Mid tibia with one well-developed anterodorsal seta, a weaker anterodorsal seta distally. Hind tibia with two dorsal preapical setae. Preapical posteroventral seta of hind tibia about as long as preapical anteroventral seta. Posterodorsal margin of hind coxa with one strong seta. *Abdomen* (Fig. 9c). Mid-dorsal depression of syntergite 1 + 2 extending on anterior half. Syntergite 1 + 2 without marginal setae. Tergite 3 with one pair of median marginal setae, tergite 4 with a row of marginal setae. Both tergites 3 and 4 without median discal setae. Tergite 5 about 7/8 as long as tergite 4. *Male terminalia* (Fig. 10d, e). Epandrium short and convex; anterior prolongation not developed. Cerci not fused medially, more or less subparallel and distally pointed in posterior view. Cercus gently bent anteriorly in lateral view. Cercus with strong setae on lateral surface and covered by thin hair-like setae. Surstylus not fused to epandrium, approximately as long as cercus. Surstylus with distal third bent postero-medially in lateral view; with short weak setae on laterodistal surface. Phallus long and straight. Epiphallus in parabasal position, well developed. Medioventral sclerite of distiphallus present; extension of dorsal sclerite of distiphallus developed; dorsolateral lobe of distiphallus well developed, with fine short hair-like setae. Pregonite large and ventrally pointed, posterior margin with a row of stout setae. Postgonite long, narrow, slightly curved. Bacilliform sclerite stick-like. Phallus apodeme robust with a well-developed phallic guide. Hypandrium well developed, medial plate of short and concave; hypandrial arms short, narrow, not fused. *Puparium*. Not preserved in examined specimens.

**Distribution** Thailand.

**Hosts** Embioptera: undescribed species (labelled with an unavailable genus name by Ross, possibly *Lobosembia* Ross, Oligotomidae) (Thailand).

**Etymology** The specific epithet *“aeratus*” means “bronze-colored”. It should be treated as a Latin adjective.

**Type material** Holotype ♂: HOLOTYPUS ♂ / *Rossimyiops* / *aeratus* sp. nov. / Badano et al. det. 2021 // Thailand: 11 km / NW Chiang Dao, / emerged 16-IV- // 1979, killed 19-IV- / 1979, E. S. Ross. / Host: *Lobembia* [unavailable genus name] n. / sp. // Collection of the California Academy of Sciences, San Francisco, Calif. [CAS] (Fig. S1 in Additional file 1). Paratype ♂: Thailand: Doi Pui, / N. of Chiangmai, / 1400 m, fly emerged / 29-Dec.-1978 / Edward S. Ross // Collection of the California Academy of Sciences, San Francisco, Calif. // odd scutellar / pattern / det: D. M. Wood, 2012 [CAS].

***Rossimyiops austrinus*** Cerretti, 2009

*Rossimyiops austrinus* Cerretti *in* Cerretti et al. 2009: 40. Type locality: Namibia, Karibib District, Tsaobismund, 22°22′40″S 15°44′58″E.

**References** Cerretti et al. [20] [taxonomic review]

**Distribution** Namibia.

**Hosts** Unknown.

***Rossimyiops djerbaensis*** Cerretti, 2009

*Rossimyiops djerbaensis* Cerretti *in* Cerretti et al. 2009: 42. Type locality: Tunisia, Djerba.

**References** Cerretti et al. [20] [taxonomic review]

**Distribution** Tunisia.

**Hosts** Unknown.

***Rossimyiops exquisitus*** (Richter, 2001)

*Persedea exquisita* Richter, 2001: 28. Type locality: Tehran (Iran).

*Mesnilomyia rufipes* Zeegers 2007: 411. Type locality: 12 km NW of Manakhah (Yemen).

**References** Cerretti et al. [20] [taxonomic review]

**Distribution** Iran, Yemen.

**Hosts** Embioptera: unidentified taxon [34].

***Rossimyiops fuscus*** **sp. nov.**

**LSID** urn:lsid:zoobank.org:act:9C37B5A7-860E-490F-A818-5F7A4A421E91.

**Diagnosis** Body length 3 mm. Inner vertical setae well developed and crossed. Parafacial, at its narrowest point approximately 1/4 width of postpedicel at mid length. Lower facial margin well visible in lateral view in front of vibrissal insertion. Pedicel brown. Postpedicel 1.5 times as long as pedicel. Arista thickened on proximal 1/4. Prementum stubby, 1.8 times as long as wide. Prosternum bare. Proepimeral seta curved downward. Wing brownish, pigmented especially on anterior part. Cell r_4 + 5_ with a petiole 1.1 times as long as postangular section of vein M_1_. Posterodorsal margin of hind coxa with one strong seta.

**Fig. 11:**
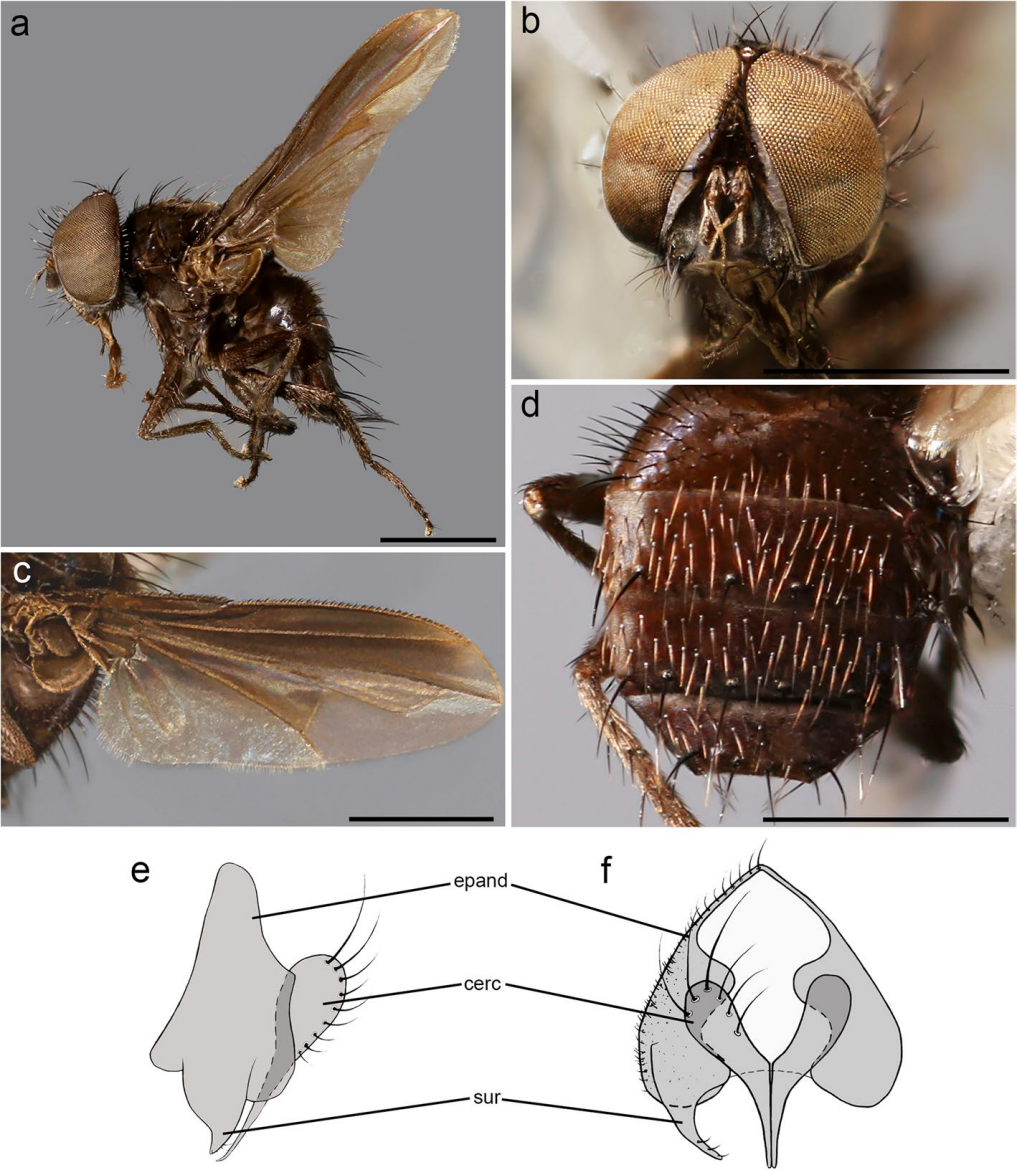
*Rossimyiops fuscus* **sp. nov**., male holotype. **a** habitus in lateral view. **b** head in frontal view. **c** wing. **d** abdomen in dorsal view. **e–f** male terminalia, **e** in lateral view, **f** in posterior view. Scale bar 1 mm

**Description (male)** Body length: 3 mm. *Color* (Fig. 11). Head black in ground color, covered with weak pruinosity. Scape, pedicel and postpedicel brown. Palpus brown. Thorax black. Presutural area without pruinosity and not showing longitudinal dark vittae. Upper and lower calypter brown. Wing brownish, pigmented especially on anterior part. Tegula and basicosta blackish brown. Wing veins brown. Legs black. Abdomen black, without pruinosity. Terminalia blackish. *Head* (Fig. 11b). Frons at its narrowest point about 1/10 as wide as compound eye in dorsal view. Outer vertical seta not distinguishable from the rest of postocular setae. Inner vertical setae well developed and crossed. Ocellar seta well developed and proclinate. Frontal setae descending to middle of pedicel. Fronto-orbital plate bare. Proclinate and reclinate orbital setae absent. Parafacial at its narrowest point approximately 1/4 of width of postpedicel at mid length. Parafacial measured ventrally at its narrowest point 1/4 of minimum distance between inner margin of compound eye and antennal insertion. Parafacial bare below lowest frontal seta. Facial ridge slightly convex, with setae above vibrissa on lower 1/4. Lower facial margin well visible in lateral view in front of vibrissal insertion. Gena about 1/10 of compound eye height. Genal dilation well developed. Postpedicel 1.5 times as long as pedicel. Arista thickened on proximal 1/4. Prementum stubby, 1.9 times as long as wide. Palpus apically enlarged. *Thorax.* Scutum with two presutural acrostichal setae; two presutural and three postsutural dorsocentral setae; two postsutural intra-alar setae separated by a distance greater than distance between first seta and suture; first postsutural supra-alar seta longer than notopleural setae. Prosternum bare. Proepimeral seta curved downward. Postpronotum with two setae. Katepisternum with two setae. Scutellum with three pairs of marginal setae (basal, subapical, apical); apical scutellar setae crossed; subapical scutellar setae shorter than apical setae. *Wing* (Fig. 11c). Costal spine not distinguishable from other costal setae. Veins R_1_ and R_4 + 5_ bare. Bend of vein M_1_ forming an obtuse angle, without stub. Section of vein M_1_ between crossveins r-m and dm-m approximately as long as section between dm-m and bend of vein M_1_. Cell r_4 + 5_ with a petiole 1.1 times as long as postangular section of vein M_1_. *Legs.* Preapical anterodorsal seta of fore tibia longer than dorsal preapical seta. Mid tibia with two anterodorsal setae. Hind tibia with two dorsal preapical setae. Preapical posteroventral seta of hind tibia about as long as preapical anteroventral seta. Posterodorsal margin of hind coxa with one strong seta. *Abdomen* (Fig. 11d). Mid-dorsal depression of syntergite 1 + 2 extending on anterior half. Syntergite 1 + 2 with two pairs of marginal setae. Tergites 3 with one pair of median marginal setae, tergite 4 with a row of marginal setae. Both tergites 3 and 4 without median discal setae. Tergite 5 about 4/5 as long as tergite 4. *Male terminalia* (Fig. 11e, f). Epandrium short and convex; anterior prolongation not developed. Cercal prongs closely abutted medially (not fused); basal two-thirds of cerci wide in posterior view, distal third strongly pointed. Surstylus approximately as long as cercus. Phallus, surstylus and hypandrial complex not examined, missing in the holotype. *Puparium*. Not preserved in examined specimens.

**Distribution** Thailand.

**Hosts** Embioptera: undescribed species (labelled with an unavailable genus name by Ross, possibly *Lobosembia* Ross, Oligotomidae) (Thailand).

**Etymology** The specific epithet “*fuscus*” means “dark”. It should be treated as a Latin adjective.

**Type material** Holotype ♂: HOLOTYPUS ♂ / *Rossimyiops* / *fuscus* sp. nov. / Badano et al. det. 2021 // Thailand: 11 km / NW Fang, high ever- / green forest, fly // emerged, 12-Mar- / 1979, E. S. Ross / Host: *Lobembia* [unavailable genus name] n. / sp. // Collection of the California Academy of Sciences, San Francisco, Calif. [CAS] (Fig. S1 in Additional file 1). Paratype ♂: Thailand: 11 km / NW Fang, high ever- / green forest, third / instar of host coll- // ected 27-Nov-1978, / fly emerged, 7-Mar- / 1979, E. S. Ross / Host: *Lobembia* [unavailable genus name] n. / sp. // Collection of the California Academy of Sciences, San Francisco, Calif. // could fit / Strongastrini [sic] / det: D. M. Wood 2012. Paratype ♂: Thailand: Doi Pui, / N. of Chiangmai, / 1400 m, fly killed / 24-Aug-1989 / Edward S. Ross // Collection of the California Academy of Sciences, San Francisco, Calif. [All in CAS.]

***Rossimyiops longicornis*** (Kugler, 1972)

*Mesnilomyia longicornis* Kugler, 1972: 108. Type locality: Ẕefat (Israel).

**References** Cerretti et al. [20] [taxonomic review]

**Distribution** Armenia, Azerbaijan, Bulgaria, Greece (including Crete, North Aegean Islands, Zakynthos), Israel, Turkey.

**Hosts** Embioptera: *Haploembia solieri* (Rambur), *Haploembia megacephala* Krauss (doubtful) and an apparently undescribed species of *Haploembia* Verhoeff (Oligotomidae) (new records).

**Material examined** 1♂: Greece: Mt. Parnis above / Athens, 1000 m / Host: *Haploembia* / *solieri*, fly matured // 24-May-1984 / Ex *Haploembia meg*- / *acephala*? / Edward S. Ross. // Collection of the California Academy of Sciences, San Francisco, Calif. 1♂: Greece: 2.5 mi. W. / of Kalavitra, 1660 m. / Peloponnese, fly // matured 14-June-1984 / Host: *Haploembia* / *solieri* / Edward S. Ross // Collection of the California Academy of Sciences, San Francisco, Calif. 1♂: Turkey: 14 km SW / of Korkuteli, 1300 / m, Host *Haploembia* // n. sp., fly emerged / 23-Jun-1984, / Edward S. Ross // Collection of the California Academy of Sciences, San Francisco, Calif. 1♀: Greece: Vergina, at / Phillip’s Place, 190 m / Host: *Haploembia* // *solieri*, fly matured / 15-Jun-1984 / Edward S. Ross // Collection of the California Academy of Sciences, San Francisco, Calif. 1♀: Turkey: 10 m. S of / Troy, Host *Haplo* / *embia solieri* fly // emerged 10-Jun-1984, Edward S. Ross // Collection of the California Academy of Sciences, San Francisco, Calif. [All in CAS.]

***Rossimyiops magnificus*** (Kugler, 1972)

*Mesnilomyia magnifica* Kugler, 1972: 105. Type locality: 'Arad (Israel).

**References** Cerretti et al. [20] [taxonomic review]

**Distribution** Egypt (Sinai), Israel, Iraq.

**Hosts** Unknown.

***Rossimyiops rutilans*** **sp. nov.**

**LSID** urn:lsid:zoobank.org:act:40BD7A46-6705-4030-9F6A-0D7A8E42D702.

**Diagnosis** Body length about 5 mm. Body overall dark brown (male) or reddish (female). Frons at its narrowest point about 1/10 (male) or 1/2 (female) as wide as compound eye in dorsal view. Inner vertical setae well developed and crossed. Gena about 1/10 of compound eye height. Postpedicel 2.2 times as long as pedicel. Arista thickened on proximal 2/5. Prosternum with two strong setae. Posterior proepimeral seta curved downward. Wing brownish on anterior surface. Vein R_4 + 5_ bare. Cell r_4 + 5_ closed at wing margin. Posterodorsal margin of coxa with 1 strong seta. Abdominal tergites 3, 4 and 5 with a narrow anterior band of pruinosity, interrupted along midline. Mid-dorsal depression of syntergite 1 + 2 extended on anterior half. Male: surstylus firmly fused to epandrium.

**Fig. 12:**
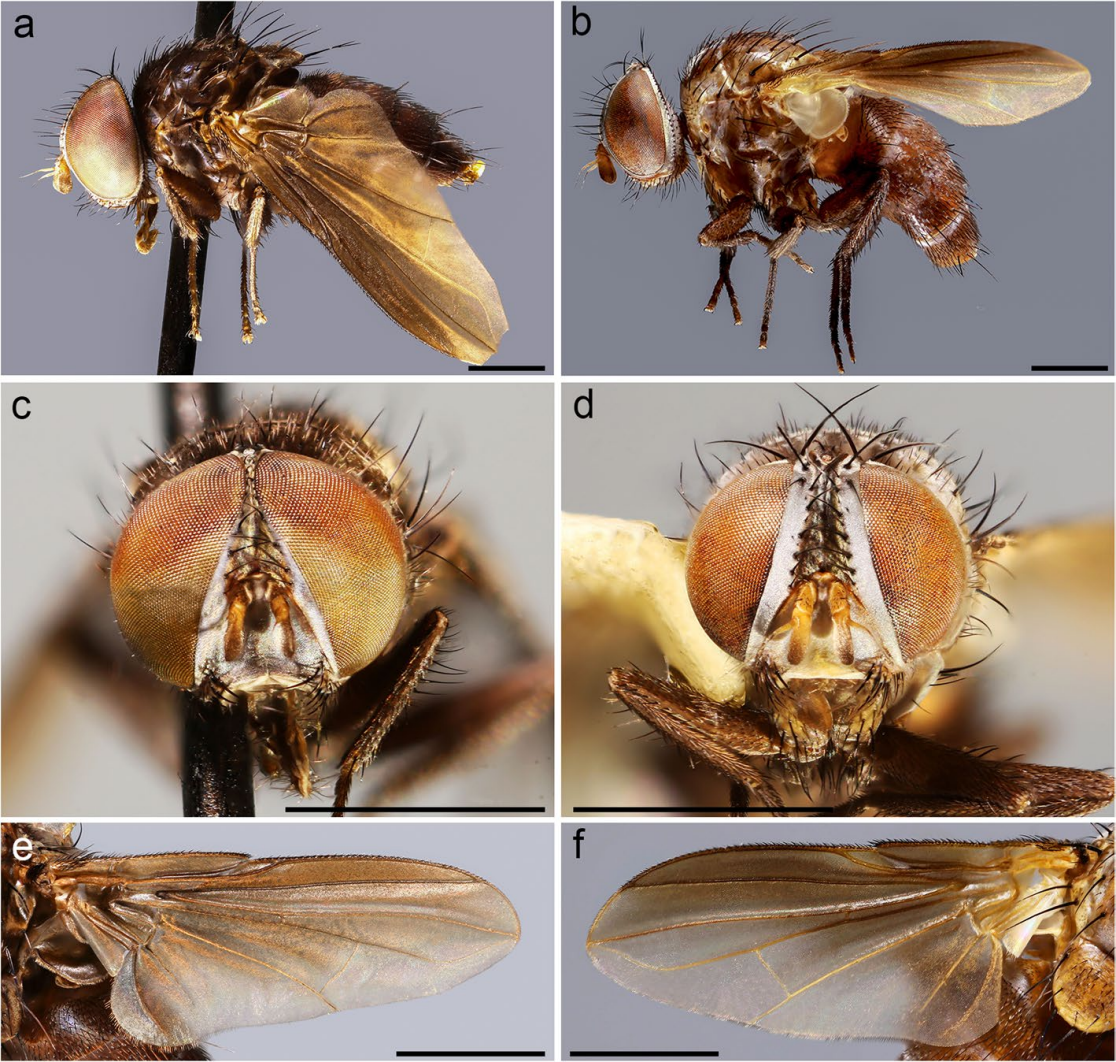
*Rossimyiops rutilans* **sp. nov.**, **a** male paratype, habitus in lateral view. **b** female paratype, habitus in lateral view. **c** male paratype, head in frontal view. **d** female paratype, head in frontal view. **e** male paratype, wing. **f** female paratype, wing. Scale bar 1 mm

**Fig. 13:**
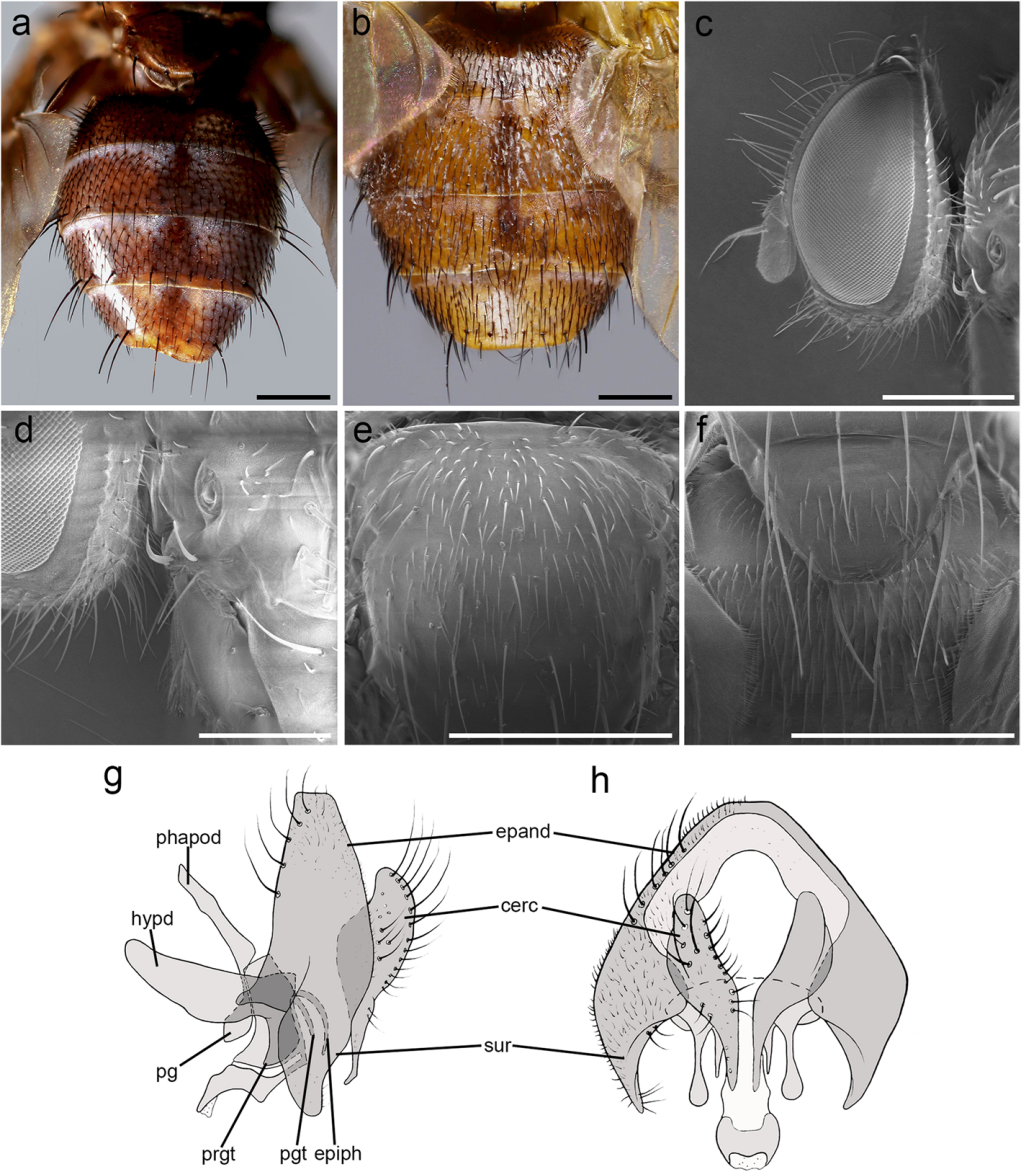
*Rossimyiops rutilans* **sp. nov.**, **a** male paratype, abdomen in dorsal view. **b** female paratype, abdomen in dorsal view. **c** male paratype, head in lateral view at SEM. **d** male paratype, thorax and posterior proepimeral seta at SEM. **e** thorax in dorsal view at SEM. **f** scutellum in dorsal view at SEM. **g–h** male terminalia, **g** in lateral view, **h** in posterior view. Scale bar 500 μm

**Description** (male, female) Body length: ca. 5 mm. *Color* (Fig. 12). Head black (male) or reddish (female) in ground color, covered with pruinosity. Scape and pedicel reddish-yellow. Postpedicel reddish-yellow, darkened at tip. Palpus reddish. Thorax brown (male) or reddish (female). Presutural area with whitish pruinosity except on 4 brown vittae (only in female). Legs brown in male, reddish in female. Upper and lower calypter light brown (male) or whitish (female). Wing brownish on anterior surface. Tegula and basicosta brown. Wing veins light brown. Scutellum brown (male) or reddish (female). Abdomen brown (male) or reddish (female). Tergite 3 and 4 with a narrow anterior band of pruinosity interrupted along midline. Terminalia brownish. *Head* (Figs. 12c, d, 13c). Frons at its narrowest point about 1/10 (male), 1/2 (female) as wide as compound eye in dorsal view. Outer vertical seta not distinguishable from the rest of postocular setae in male and well developed in female. Inner vertical setae well developed and crossed. Ocellar seta well developed and proclinate. Frontal setae descending to the upper margin of pedicel. Fronto-orbital plate bare. Reclinate orbital setae absent in male, one or two in female (asymmetric in female paratype). Parafacial, at its narrowest point approximately 1/2 (male), 3/5–4/5 (female) of width of postpedicel at mid length. Parafacial measured ventrally at its narrowest point 1/4 (male), 2/5 (female) of minimum distance between inner margin of compound eye and antennal insertion. Parafacial bare below lowest frontal seta. Facial ridge slightly straight with erect setae above vibrissa on lower 1/5. Lower facial margin not visible in lateral view in front of vibrissal insertion. Gena about 1/10 of compound eye height. Genal dilation well developed. Postpedicel 2.2 times as long as pedicel. Arista thickened on proximal 2/5. First aristomere very short, no longer than wide. Second aristomere as long as wide*.* Prementum 3 times as long as wide. Palpus apically enlarged. *Thorax* (Fig. 13d-f). One presutural acrostichal setae; two presutural and three postsutural dorsocentral setae; two postsutural intra-alar setae separated by a distance about equal to distance between first seta and suture; first postsutural supra-alar seta longer than notopleural setae. Prosternum with two strong setae. Posterior proepimeral seta curved downward (Fig. 13d). Postpronotum with two setae. Katepisternum with two setae. Scutellum with three pairs of marginal setae (basal, subapical, apical) (Fig. 13f); apical scutellar setae crossed; subapical scutellar setae as long as apical setae. *Wing* (Fig. 12e, f). Costal spine not distinguishable from other costal setae. Vein R_1_ and R_4 + 5_ bare. Bend of vein M_1_ forming an obtuse angle. Section of vein M_1_ between crossveins r-m and dm-m approximately as long as section between dm-m and bend of vein M_1_. Cell r_4 + 5_ closed at wing margin. *Legs.* Preapical anterodorsal seta of fore tibia visibly longer than dorsal preapical seta. Mid tibia with two anterodorsal setae. Hind tibia with two dorsal preapical setae. Anteroventral surface of fore coxae completely bare. Preapical posteroventral seta of hind tibia as long as preapical anteroventral seta. Posterodorsal margin of hind coxa with one strong seta. *Abdomen* (Fig. 13a, b). Mid-dorsal depression of syntergite 1 + 2 extending on anterior half. Syntergite 1 + 2 without marginal setae. Tergites 3 with one pair of median marginal setae, tergite 4 with a row of marginal setae. Both tergites 3 and 4 without median discal setae. Tergite 5 as long as tergite 4. *Male terminalia* (Fig. 13g, h). Epandrium very short and convex; anterior prolongation not developed. Cerci not fused medially and cercal prongs standing widely apart, distally pointed in posterior view. Cercus pointed and apically curved anteriorly in lateral view. Basal half of cercus with strong erect setae on apical dorsal surface. Surstylus broad and fused to epandrium, longer than cercus. Surstylus with short weak setae on laterodistal surface. Phallus short. Epiphallus in parabasal position, very narrow and curved. Medioventral sclerite of distiphallus present; extensions of dorsal sclerite of distiphallus not developed; dorsolateral lobe of distiphallus not developed. Pregonite broad lobe-like. Postgonite very narrow and almost straight. Bacilliform sclerite not differentiated. Phallus apodeme robust with a well-developed and wide phallic guide, concave in anterior view. Hypandrium well developed, medial plate concave, hypandrial arms narrow and firmly fused postero-medially, entirely encircling base of phallus. *Puparium*. Sub-cylindrical in shape. Roundly convex anteriorly, tapering toward distal fourth and ending in two small subconical spiracular projections (spiracular openings not visible at 90x magnification). Reddish in ground color, generally smooth but covered with micro-spines. One of the puparia was covered with remains of host.

**Distribution** Myanmar.

**Hosts** Embioptera: undescribed species (labelled with an unavailable genus name by Ross) (family not given) (Myanmar) and unidentified species of *Ptilocerembia* Friederichs (Ptilocerembiidae) (Myanmar).

**Type material** Holotype ♂: HOLOTYPUS ♂ / *Rossimyiops* / *rutilans* sp. nov. / Badano et al. det. 2021 // Burma: Maymyo, / 3538′, Pupated 8- / Jan-1979, pupated / 9 Jan-1979, emerged / 23-Jan-1979, died // 26-Jan-1979. / collected in native / forest. Host is prob. / *Heoembia* [unavailable genus name] n. sp. / Edward S. Ross // Collection of the California Academy of Sciences, San Francisco, Calif. [CAS; puparium of holotype is pinned separately and labelled as the holotype] (Fig. S1 in Additional file 1). Paratype ♂: Burma: Maymyo, 3538′, Pupated 8- / Jan-1979, pupated / 8 Jan-1979, emerged / 22-Jan-1979, died // 25-Jan-1979, E. S. / Ross, collected in / native forest. Host is / prob. *Heoembia* [unavailable genus name] n. / sp. // Collection of the California Academy of Sciences, San Francisco, Calif. [Puparium of paratype male is pinned separately and labelled as the paratype]. Paratype ♀: Burma: Maymyo, / 3538′, emerged V-7- / 1979, died V-9- / 1979, E. S. Ross, // culture collected on / tree trunks in patch / of native forest. / Host: *Ptilocerembia* // Collection of the California Academy of Sciences, San Francisco, Calif. [All in CAS.]

**Etymology** The specific epithet *rutilans* (i.e., present participle of the Latin verb *rutilō*) means “that shines in red”. It should be treated as a Latin adjective.

***Rossimyiops subapertus*** (Herting, 1983)

*Mesnilomyia subaperta* Herting 1983: 5. Type locality: Anbar-Abad (Iran).

**References** Cerretti et al. [20] [taxonomic review]

**Distribution** Iran, Israel, Turkmenistan.

**Hosts** Unknown.

***Rossimyiops whiteheadi*** Mesnil, 1953

*Rossimyiops whiteheadi* Mesnil, 1953: 145. Type locality: Grahamstown (South Africa).

**References** Cerretti et al. [20] [taxonomic review]

**Distribution** South Africa (Eastern Cape).

**Hosts** Embioptera: unidentified species of *Apterembia* Ross (Embiidae).

### **Identification key to*Rossimyiops*species**

Modified from Cerretti et al. [20].

1. Wing vein M_1_ not reaching wing margin (i.e., ending freely in wing membrane ....................................................................undescribed species #1 from Nigeria (CNC)

- Wing vein M_1_ reaching wing margin (Figs. 9b, 12e, f) or fused to vein R_4 + 5_, so that cell r_4 + 5_ is petiolate (e.g., Fig. 11c) ......................................................................2
  2. Wing cell r_4 + 5_ open (Fig. 9b), closed at wing margin (Fig. 12e, f), or very short petiolate. Scutellum with three marginal setae (Fig. 10c). Wing vein R_4 + 5_ bare (Fig. 9b) or with one basal seta .......................................3
- Wing cell r_4 + 5_ long petiolate (Fig. 11c). Scutellum with two or three marginal setae. Base of R_4 + 5_ bare .............6
  3. Inner vertical setae crossed (Fig. 12c, d). Posterodorsal margin of hind coxa with one strong seta. Oriental Region .............................................................................4
- Inner vertical setae subparallel. Posterodorsal margin of hind coxa bare. Other regions ...................................5
  4. Prosternum with two strong setae (one each side). Posterior proepimeral seta curved downward (Fig. 13d). Abdominal tergites 3 and 4 with a narrow anterior band of pruinosity interrupted along midline (Fig. 13a, b). Male: epandrium and surstylus firmly fused (Fig. 13g, h). Female body reddish in ground color ......................................................*R. rutilans* sp. nov.
- Prosternum bare. Posterior proepimeral seta not as above. Tergite 3 and 4 without pruinosity (Fig. 9c). Male: epandrium and surstylus not fused (Fig. 10d, e). Female unknown ...........................*R. aeratus* sp. nov.
  5. Lower facial margin not visible in lateral view in front of vibrissal insertion. Prementum 2.0–2.3 times as long as its diameter at mid length. Fore tibia with two posterior setae. Mid tibia with one anterodorsal seta. Presutural area of scutum with lateral longitudinal dark vittae very small, not reaching the transverse suture posteriorly. Abdomen shiny black, without pruinosity. Male: cercus and surstylus stout; cercus apically rounded in posterior view. Female: lateral vertical seta not differentiated from the postocular row ...............................................*R. subapertus* (Herting)
- Lower facial margin well visible in lateral view, anterior to vibrissal angle. Prementum very elongated, 6–10 times as long as wide. Fore tibia with one posterior seta. Mid tibia with two anterodorsal setae. Presutural area with lateral longitudinal dark vittae broad, clearly reaching the transverse suture posteriorly. Abdominal tergites 3–5 each with a narrow anterior band of whitish pruinosity. Male: cercus and surstylus not as above; combined cerci sub-triangular in posterior view, apically pointed. Female: lateral vertical seta well developed and differentiated from the postocular row ........................*R. whiteheadi* Mesnil
  6. Scutellum with three pairs of marginal setae (e.g., Fig. 10c). Thoracic presutural area without whitish pruinosity and not showing longitudinal dark vittae. Cell r_4 + 5_ without petiole or with a petiole about 0.7–1.0 times as long as postangular section of vein M_1_ ............................7
- Scutellum with two pairs of marginal setae. Thoracic presutural area with whitish pruinosity except on three longitudinal dark vittae. Cell r_4 + 5_ with petiole about 0.3–1.3 times as long as postangular section of vein M_1_ .................................................................................9
  7. Proepimeral seta curved downward (e.g., Fig. 13d). Posterodorsal margin of hind coxa with one strong seta. Oriental Region ............................*R. fuscus* sp. nov.
- Proepimeral seta, if present, not curved downward. Posterodorsal margin of hind coxa bare. Other regions ........8
  8. Parafacial at its narrowest point 2 times as wide as maximum diameter of arista. Thorax (including scutellum), coxae, femora, and palpus yellow. Abdominal syntergite 1 + 2 and tergite 3 yellow at least anteroventrally; tergites 4 and 5 usually black (at least dorsally). Wing hyaline. Ventral seta of mid tibia longer than maximum diameter of mid tibia ........................*R. exquisitus* (Richter)
- Parafacial at its narrowest point about 3–4 times as wide as maximum diameter of arista. Thorax (including scutellum) and femora black, coxae varied from black to reddish, palpus basally yellowish-brown, shading into black distally. Abdomen black. Wing slightly smoky anteromedially and along veins. Ventral seta of mid tibia weak and shorter than maximum diameter of mid tibia ...................*R. djerbaensis* Cerretti
  9. Frons very narrow, no more than 0.13 of compound eye in dorsal view (usually less); proclinate orbital setae absent; frontal vitta very narrow, practically indistinct anterior to fore ocellus [males] ........................................10

[male of *R. austrinus* unknown]

- Frons at least as wide as compound eye in dorsal view (usually more), with two or more proclinate orbital setae, frontal vitta not as above [females] ..................12
  10. Face flat, ventral facial margin not visible in lateral view. Prementum about 2–3 times as long as its diameter. Postpedicel 2.5–3.0 times as long as pedicel. Wing hyaline. Section of vein M_1_ between crossveins r-m and dm-m distinctly shorter than section between dm-m and bend of vein M_1_. Cell r_4 + 5_ with petiole more than 0.5 times as long as postangular section of vein M_1_ .......................*R. longicornis* (Kugler)
- Ventral facial margin well visible in lateral view, anterior to vibrissal angle. Prementum about 4–5 times as long as its diameter. Postpedicel 1.7–2.2 times as long as pedicel. Wing hyaline or brownish anteriorly. Section between crossvein r-m and dm-m approximately as long as section between dm-m and bend of vein M_1_. Cell r_4 + 5_ with petiole 0.3–0.5 (rarely more) times as long as postangular section of vein M_1_ ..................11
  11. Wing anteriorly brownish. Postpedicel 1.9–2.2 times as long as pedicel. Body length: 2.5–3.5 mm ...........................................................................*R. achilleae* (Kugler)
- Wing hyaline. Postpedicel 1.7–1.9 times as long as pedicel. Body length: 4.5–6.0 mm ........*R. magnificus* (Kugler)
  12. Postpedicel 1.0–3.1 times as long as its diameter. Wing from hyaline to brown ........................................13
- Postpedicel 5 times as long as its diameter. Wing hyaline .........undescribed species #2 from Nigeria (CNC)
  13. Postpedicel as long as pedicel. Fronto-orbital plate with dark vitta really wide on medial margin, between row of proclinate orbital setae and compound eye. Calypter whitish. Wing hyaline, slightly yellowish .........................................................................undescribed species #3 from Nigeria (CNC)
- Postpedicel 1.7–3.1 times as long as pedicel. Wing from hyaline to brown. Fronto-orbital plate not as above. Calypter from white to dark brown ................14
  14. Face flat, ventral facial margin not visible in lateral view. Postpedicel 2.7–3.1 times as long as pedicel. Prementum about 2–3 times as long as its diameter. Wing from white to smoky anterodistally. Calypter varied from brownish with a slightly darker rim to evenly dark brown. Halter yellow to light brown .........................*R. longicornis* (Kugler)
- Ventral facial margin visible in lateral view, anterior to vibrissal angle. Postpedicel about 1.7–2.5 times as long as pedicel. Prementum about 4–5 times as long as its diameter. Wing not as above. Calypters white to yellowish. Halter yellow to black ..................................15
  15. Frons about 0.5 times as wide as compound eye in dorsal view. Parafacial, in lateral view about 0.5–0.6 as wide as postpedicel. Fronto-orbital plate with dark stripe on its medial margin, between the row of proclinate orbital setae and setae and the frontal vitta. Ventral facial margin well visible in lateral view, anterior to vibrissal angle, protruding by about the distal width of antennal pedicel. Halter black. Wing membrane slightly infuscate anteriorly. Coxae black. Abdomen shiny black, without whitish pruinosity. Postpedicel 2.0–2.5 times as long as pedicel ......................................................*R. austrinus* Cerretti
- Frons at least 0.6 times as wide as compound eye in dorsal view. Parafacial, in lateral view, 0.7–1.0 times as wide as postpedicel. Fronto-orbital plate entirely and evenly covered with whitish pruinosity. Ventral facial margin not so strongly protruding. Halter yellow to dark brown. Coxae light brown, red to yellowish. Abdomen shiny black to entirely covered with microtrichia. Postpedicel 1.68–2.47 times as long as pedicel .....................................16
  16. Postpedicel 1.7–2.5 times as long as pedicel. Body length: 2.5–3.5 mm .........................*R. achilleae* (Kugler)
- Postpedicel 1.7–1.9 times as long as pedicel. Body length: 4.5–6.0 mm .....................*R. magnificus* (Kugler)

**Remarks** The three new species described here, *R. aeratus* **sp. nov.**, *R. fuscus* **sp. nov.** and *R. rutilans* **sp. nov.**, represent the first records of *Rossimyiops* from the Oriental Region. The new species differ from their congeners by the presence of one strong seta on the posterior margin of the hind coxa and inner vertical setae crossed.

Our report of five specimens of *R. longicornis* (Kugler) that emerged from webspinners reared by Ross confirm the association of *Rossimyiops* with webspinners, an interaction previously known only for *R. exquisitus* and *R. whiteheadi* [20]. Cerretti et al. [20] and Kugler [32] remarked on the high intraspecific variability of *R. longicornis* and we also observed this in the specimens we examined.

## Discussion

Only two insect orders include parasitoids of webspinners: Diptera and Hymenoptera. These two hyperdiverse clades account for the vast majority of insect parasitoids, with the latter much more diverse in this respect. The parasitism of webspinners by Hymenoptera has evolved independently at least three times: once in the small chrysidoid family Sclerogibbidae, the members of which are obligate ectoparasitoids of embiopteran nymphs (the females are ant-like and wingless and able to smoothly maneuver through the serpentine tunnels of their hosts) [41, 42]; once in *Sericobracon* Shaw (Braconidae), in which one species (and possibly another) is an endoparasitoid of the clothodid *Antipaluria urichi* (Saussure) [43]; and at least once in the Scelionidae (genera *Embidobia* Ashmead, *Palaeogryon* Masner and *Embioctonus* Masner), which include egg endoparasitoids of webspinners [44–46]. The evolutionary path that led to the exploitation of webspinners in each of these wasp lineages is still unclear. Engel and Grimaldi [47] hypothesized that sclerogibbids were originally beetle parasitoids, although their abundance and diversity in Cretaceous amber may instead suggest that they had already exploited webspinners or related polyneopterans in the Mesozoic [48].

Parasitoids of webspinners within Diptera evolved only in the Tachinidae, the largest and most successful of all dipteran lineages of endoparasitoids. Recent reconstructions of the evolution of host preferences in this family suggest that the last common ancestor of tachinids likely developed on soil-dwelling invertebrates, and the clade later radiated and diversified on various phytophagous insect lineages (e.g., larval lepidopterans and coleopterans, and adult hemipterans) through a series of host shifts [29, 49]. Sometimes host shifts involved distantly related and/or ecologically diverging host groups, e.g., *Loewia* Egger (and relatives) and *Spilochaetosoma* Smith apparently switched to chilopods and scorpions from lepidopteran-associated ancestors [28]. A sudden host spillover may best explain the parasitism of webspinners by species of the four distantly related tachinid genera discussed herein. Species of each of these genera exploited webspinners independently and presumably did so through a shift from a host that shared ecological characteristics and/or behavioral traits with them. Interestingly, these tachinids all practice an indirect oviposition strategy, i.e., they do not lay eggs directly on (or into) their host’s body [34, 50]. They instead follow visual and chemical cues to locate the microhabitat of the host and lay eggs in places where a host may pass by. These cues include food remains, shelters, odors or other environmental features that unveil the presence of a potential host [51]. This hunting strategy can lead to the chance parasitization of non-target insects that are occasionally repeatedly successful, giving rise to new host associations and trophic interactions. Embiopteran and lepidopteran larvae, although phylogenetically very far apart, both produce silky structures (e.g., tunnels, cases, cocoons), suggesting that this cue could be used by parasitoids to help locate them. Remarkably, most of tachinids specialized on webspinners appear to be grouped with taxa developing on Lepidoptera. For example, *Phytomyptera* species often attack concealed larvae of micromoths (e.g., Pterophoridae, Tineidae and Tortricidae) [28, 29]. *Phytomyptera woodi* is the only known species of its genus with a non-lepidopteran host. Minthoini also include species developing on concealed larvae of Lepidoptera, however hosts are unknown for most of the species, hinting at possibly unusual hosts for many of them. Graphogastrines and minthoines lay membranous eggs ready to hatch, and the planidial larvae actively seek for hosts. Members of the Goniini reach their hosts in a different way. They lay tiny “microtype” eggs on the food of their hosts that are ingested by the feeding host. The eggs hatch in the gut and the first instar larvae migrate into the host haemocoel to complete development. As a rule, goniines parasitize phyllophagous caterpillars and, more rarely, sawfly larvae by laying their eggs along leaf margins, in particular those which have been chewed by hosts. To our knowledge, only a few goniines have switched to non-leaf feeders. These include several species of the genera *Pexopsis* Brauer & Bergenstamm and *Erythrocera* Robineau-Desvoidy, and the species *Manola xenocera* Richter and *Masistyloides kononenkoi* Richter, which all develop in adult beetles, *Arama gobica* Richter which develops in cockroaches [29], *Ocytata pallipes* (Fallén) which is a parasitoid of earwigs, and several species of *Allophorocera* Hendel which develop in crane fly larvae [29, 52]. Despite the lack of detailed information about deposition strategies, these goniines presumably still lay their microtype eggs on the food of their non-leaf feeder hosts. What factors were involved in these shifts in deposition strategy is unknown. Even more of a mystery is the parasitization of wood-dwelling beetle larvae by members of the goniine genus *Pseudalsomyia* Mesnil [53] because the host larvae apparently do not leave their tunnels. This situation is the most comparable to that of the webspinner parasitoids of the goniine genera *Perumyia* and *Embiophoneus*, which also attack concealed, non-phyllophagous hosts that usually do not leave their shelters. The discovery of the trophic strategies of any of these aberrant goniines may shed light on the evolutionary path that has led to these bizarre host shifts in this megadiverse tribe.

## Conclusions

Tachinids shifted to webspinners at least four times: twice in the huge tribe Goniini (*Embiophoneus* and *Perumyia*), probably once in the graphogastrine genus *Phytomyptera*, and once in the minthoine genus *Rossimyiops*. This specialization likely evolved in each lineage from ancestors sharing similar habits such as attacking silk-protected or concealed hosts or searching microhabitats like those occupied by webspinners.

## Methods

The dissection of male terminalia was carried out following the protocol described in detail by O’Hara [54]. Digital images of external morphology were taken partly using a Canon EOS 6D camera equipped with Canon Photo lens MP-E 65 mm 1:2.8 and processed by Canon Digital Photo Professional (Canon: Ōta, Tokyo, Japan), Combine ZM by Alan Hadley and GIMP 2.10.4 by Alexandre Prokoudine; partly using a scanning electron microscope Dualbeam FIB/SEM Helios NanoLab 600 FEI Company (FEI Company: Hillsboro, Oregon, USA) and processed by its software. Illustrations of male terminalia were produced by using a camera lucida and the images edited and colored in GIMP 2.10.4.

Terminology of external morphology follows Cumming & Wood [55], measurements and ratios of the head follow Cerretti [18]. Label data are reported verbatim (scientific names are given in italics as prescribed by ICZN code [56]), with the symbol “/” marking the end of each line and “//” marking the end of each label.

This publication and its nomenclatural acts have been registered in ZooBank “http://zoobank.org/”, LSID: urn:lsid:zoobank.org:pub:62FDC19C-3D03-48F9-B92F-C8CDCFB71D3B.

Abbreviations for depositories cited in this work are as follows: CAS – California Academy of Sciences, San Francisco, California, USA; CNC – Canadian National Collection of insects, arachnids and nematodes, Ottawa, Canada; MZUR – zoological museum, Sapienza University, Rome, Italy

Data labels of the holotype specimens are shown as Additional file 1.

## Supplementary Information

**Additional file 1:** **Figure S1** Holotypes labels.

## Data Availability

All relevant data are available in the main text.

## Abbreviations

acroph: Acrophallus
bac scl: Bacilliform sclerite
cerc: Cercus
dll: Lateroventral lobe of distiphallus
dlp: Lateroventral projection of distiphallus
distph: Distiphallus
epand: Epandrium
epiph: Epiphallus
hypd: Hypandrium
Pg: Phallic guide
pgt: Postgonite
phapod: Phallus apodeme
pregt: Pregonite
sur: Surstylus
