# Supplementary figures and images for: A world review of the bristle fly parasitoids of webspinners
Source: BMC Zool. 2022 Jul 4;7:37. doi: 10.1186/s40850-022-00116-x (PMC10127400; doi:10.1186/s40850-022-00116-x)

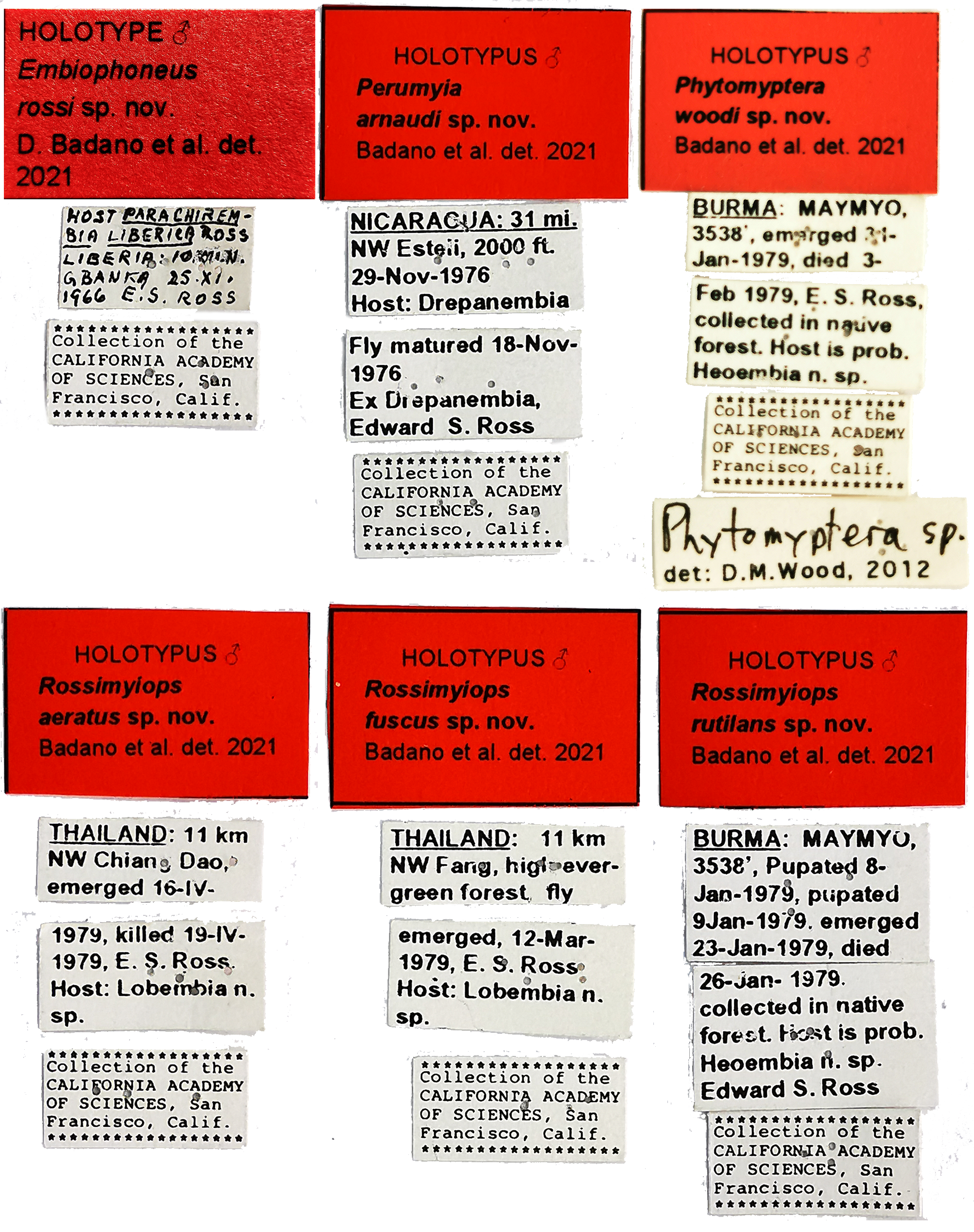

Supplement: Supplementary file 1 — Additional file 1: Figure S1 Holotypes labels. [file 40850_2022_116_MOESM1_ESM.tif]
